# Molecular characterisation of the trafficking rescue of defective ABCB4 variants by roscovitine analogues

**DOI:** 10.1038/s41598-026-39840-6

**Published:** 2026-02-25

**Authors:** Manon Banet, Veronica Crespi, Jonathan Elie, Yosra Riahi, Mounia Lakli, Elodie Mareux, Emmanuel Gonzales, Emmanuel Jacquemin, Laurent Meijer, Martine Lapalus, Florent Di Meo, Thomas Falguières

**Affiliations:** 1 Inserm, Physiopathogénèse et Traitement des maladies du foie, UMR_S 1193, FHU Hepatinov, Université Paris-Saclay, 91400 Orsay, France; 2 Inserm, Pharmacology and Transplantation, UMR 1248, Centre de Biologie et Recherche en Santé, Université de Limoges, 87000 Limoges, France; 3 ManRos Therapeutics & Perha Pharmaceuticals, Hôtel de Recherche, Presqu’île de Perharidy, 29680 Roscoff, France; 4 Assistance Publique - Hôpitaux de Paris, Paediatric Hepatology and Paediatric Liver Transplant Department, Reference Center for Rare Paediatric Liver Diseases, FILFOIE, ERN RARE LIVER, Faculté de Médecine Paris-Saclay, CHU Bicêtre, 94270 Le Kremlin-Bicêtre, France; 5 Inserm US042, CNRS UAR2015, BISCEm, Université de Limoges, CHU Limoges, 87000 Limoges, France

**Keywords:** ABC transporters, Bile secretion, Targeted pharmacotherapy, Drug candidates, Molecular modelling

## Abstract

ABCB4 is expressed at the canalicular membrane of hepatocytes and is responsible for the secretion of phosphatidylcholine into bile. Genetic variations of ABCB4 are associated with rare cholestatic liver diseases, the most severe being progressive familial intrahepatic cholestasis type 3, for which most of patients require liver transplantation before adulthood. Therefore, the development of alternative pharmacotherapies is urgently needed. We have previously shown that structural analogues of roscovitine rescue the maturation, localisation and indirectly the function of class II intracellularly-retained ABCB4 variants. Nevertheless, in order to develop molecules with better benefit/toxicity ratios, new structural analogues of roscovitine were tested in cell models as potential correctors of three class II ABCB4 variants. We show here that nine roscovitine analogues are able to significantly rescue the maturation and canalicular localisation of these ABCB4 variants. Three of these analogues were also able to partially restore the transporter-mediated activity of ABCB4 variants, while being less inhibitory of wild type ABCB4 function than roscovitine itself. In addition, ensemble docking calculations suggest that the selected roscovitine analogues may directly interact with wild type or mutated ABCB4. Our results represent a new step towards the identification of pharmacological correctors for ER-retained variants of the ABCB4 transporter.

**Supplementary Information:**

The online version contains supplementary material available at 10.1038/s41598-026-39840-6.

## Introduction

Bile secretion is an essential function of the liver, necessary for the digestion of dietary fats and the elimination of xenobiotics and endogenous metabolites. This function depends mainly on the activity of ABC (ATP-binding cassette) transporters, which are transmembrane proteins able to bind and hydrolyse ATP to fulfil their biological functions^1,2^. Among these transporters specifically located at the canalicular membrane of hepatocytes, the ABC transporter subfamily B member 4 (ABCB4) enables the secretion of phosphatidylcholine (PC) into the bile by flopping it from the inner leaflet to the outer leaflet of the plasma membrane^3,4^. Together with bile salts and cholesterol, which are co-secreted by ABCB11 and ABCG5/G8, respectively, PC forms mixed micelles in bile with a tightly-controlled ternary equilibrium^5^. An imbalance in either of these elements may lead to the formation of cholesterol crystals and gallstones in the biliary tract, leading to cholestasis, as well as loss of protection against the detergent effects of free bile salts on the biological membranes of the biliary tree^6^.

Genetic variations in the *ABCB4* locus are associated with several cholestatic diseases, the most severe and rare form of them being progressive familial intrahepatic cholestasis type 3 (PFIC3)^7^. This rare autosomal recessive disease appears during the first months or years of life and often progresses to cirrhosis and liver failure^8,9^. First-line treatment is based on the administration of ursodeoxycholic acid (UDCA), a bile acid with low hydrophobicity, which improves the condition of patients with mild forms of this cholestatic disease^10^. However, this treatment is not, or not sufficiently, effective in patients with the most severe forms of these diseases, who most often require liver transplantation^8,11,12^. There is therefore a need to develop pharmacological alternatives.

To date, more than 1500 genetic variations of the *ABCB4* locus have been reported in patients, potentially affecting more than 75% of the 1279 amino acids of the protein (https://gnomad.broadinstitute.org/; https://bravo.sph.umich.edu/; http://abcm2.hegelab.org/—accessed on 12/07/2025), most of them being missense variations. These ABCB4 variants can be affected at different molecular/cellular levels, leading to their classification in four classes: expression (class I), maturation and exit from the endoplasmic reticulum (ER) (class II), PC secretory function (class III) or plasma membrane stability (class IV)^13–15^. This classification has a fundamental role for a targeted pharmacotherapy approach since molecular/cellular defects of ABCB4 variants have to be first characterised before considering the use of specific drugs in a personalised therapeutic strategy. Furthermore, molecular mechanisms of ABCB4 variant defects remain elusive since the structural impact of class II, III and IV variants still require investigation.

Focusing on traffic-defective ABCB4 variants (class II), many efforts have been made to develop new therapies in order to avoid, or at least delay, liver transplantation. In the frame of repurposing strategies, it has been shown that roscovitine, a trisubstituted purine derived from starfish oocytes^16^, can correct the intracellular localisation and the channel activity of the F508del variant of cystic fibrosis transmembrane conductance regulator (ABCC7/CFTR)^17^, and also rescues the maturation and canalicular targeting of class II ABCB4 variants^18^. However, roscovitine is cytotoxic and inhibits wild type (WT) ABCB4 (ABCB4-WT) activity^18^. Therefore, structural analogues of this molecule were synthesised and we showed that they were able to partially restore the maturation, localisation and indirectly the PC secretory function of several ER-retained ABCB4 variants but they also significantly inhibit the activity of the WT transporter^18^, as observed with other families of molecules^19,20^**.**

Here, we screened 53 newly synthesised roscovitine analogues in order to find molecules with the best benefit/inhibition ratio. We showed that nine roscovitine analogues correct the maturation and the canalicular localisation of three ER-retained ABCB4 class II variants (I541F, L556R and I490T) and more importantly three of them partially rescue the residual secretory function of these ABCB4 variants. The present study also takes advantage of molecular simulations to: *i)* provide insights into the structural impact of the selected class II variants; and *ii)* better understand the binding modes of these molecules by investigating potential direct interactions with WT and ABCB4 variants.

## Results

### Identification of roscovitine analogues as potential correctors of traffic-defective ABCB4 variants

**Fig. 1:**
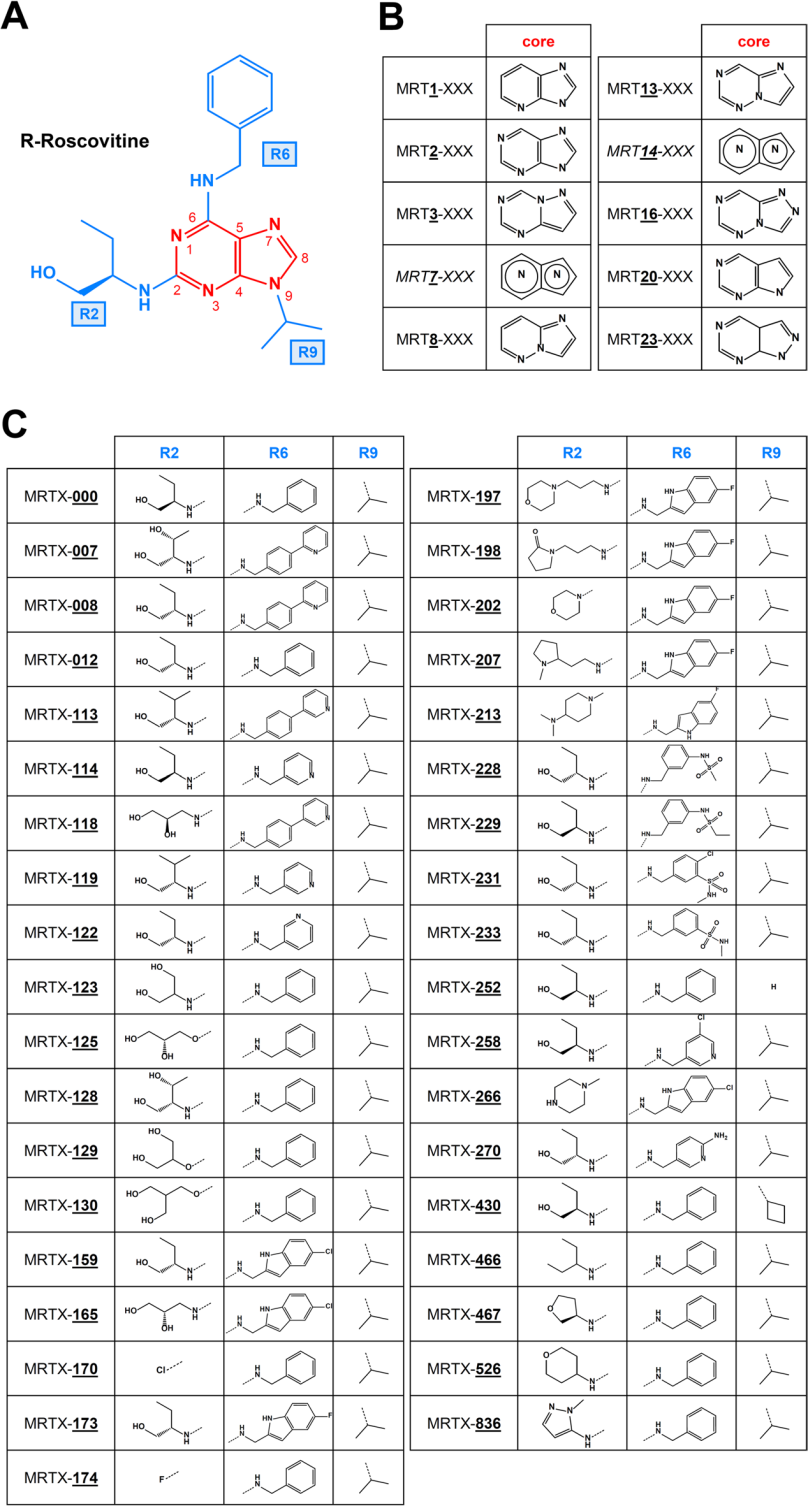
Roscovitine analogues used in this study. (**A**) The chemical structure of R-roscovitine is shown with its purine core (red) and its three substituents R2, R6 and R9 (blue). (**B**,**C**) Modification of the core (**B**) and substituents (**C**) from A of the roscovitine analogues used in this study. Note that for intellectual property reasons (ongoing patent pending), MRT7-XXX and MRT14-XXX cores have been replaced by a generic structure.

**Fig. 2:**
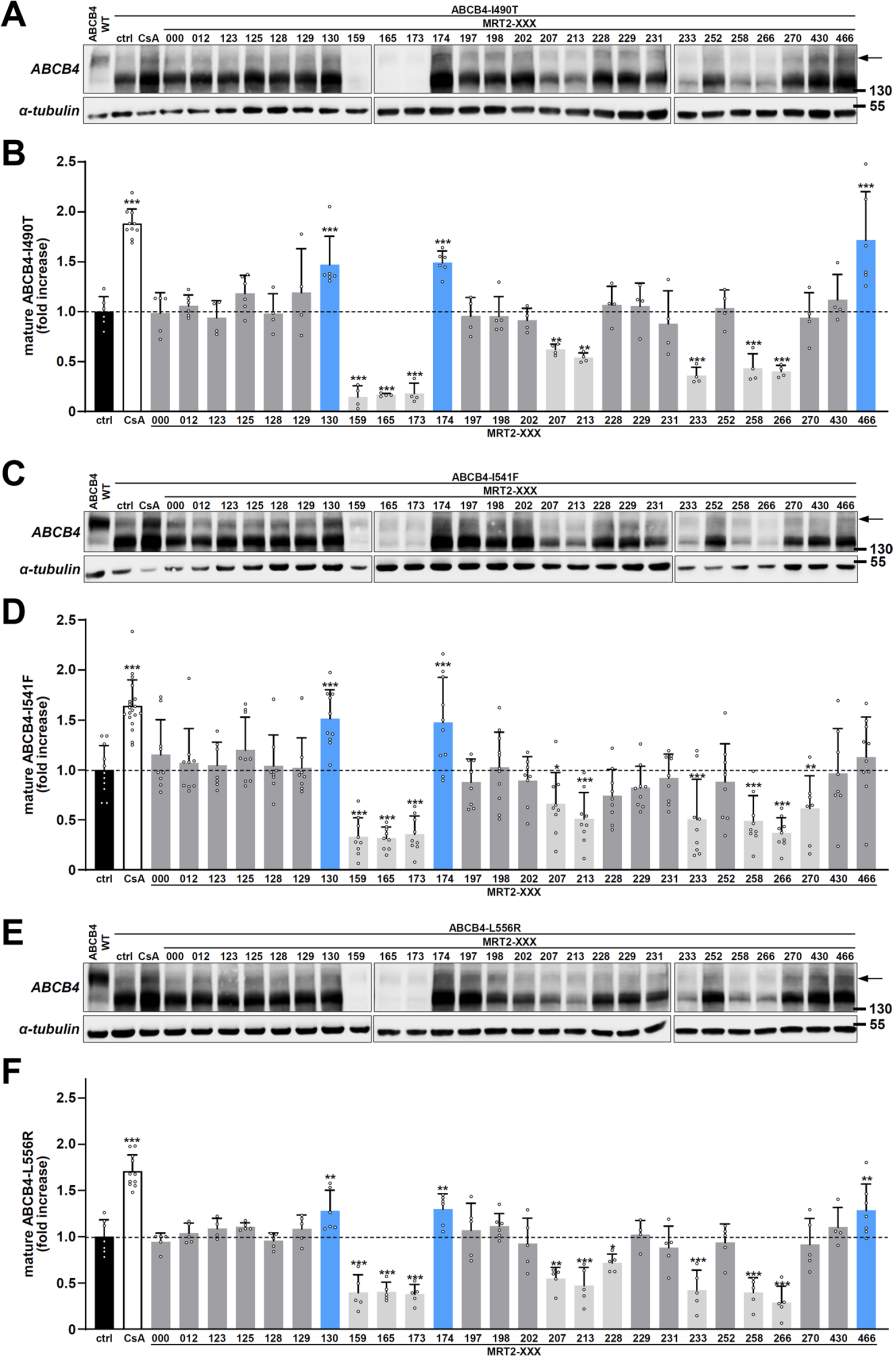
Maturation analysis of class II ABCB4 variants after treatment with roscovitine analogues with modified substituents. (**A**) HEK cells transiently expressing ABCB4-I490T were treated for 16 h with vehicle (ctrl, DMSO), 10 µM cyclosporin A (CsA) or 10 μM of the indicated roscovitine analogues (series MRT2-XXX, see Fig. 1). Cell lysates were prepared and analysed by immunoblot using the indicated antibodies. ABCB4-WT is shown as reference. The arrow indicates the mature form of ABCB4. Cropped images from full immunoblots are delineated. Molecular weight markers (in kDa) are indicated. (**B**) Densitometry analysis of A. The amount of mature form of ABCB4 variants was quantified, normalised to the α-tubulin levels and then expressed as a fold-increase of the means for vehicle-treated cells (ctrl). The dashed line indicates the reference value for the control condition. Means (± SD) of at least four independent experiments per condition are shown. Blue bars indicate positive statistical significance while light grey bars indicate negative statistical significance, by opposition to dark grey bars (no significance). Ctrl: control (vehicle); CsA: cyclosporin A. (**C**–**F**) Same as (**A**,**B**) with HEK cells expressing ABCB4-I541F (**C**,**D**) or ABCB4-L556R (**E**,**F**). (**A**,**C**,**E**) panels are representative of at least four independent experiments per condition. Full immunoblots are shown in Supplementary Fig. 4.

Based on our previous study on a small number of roscovitine analogues with only modified substituents^18^, we extended our pharmacotherapy database beyond R-roscovitine (MRT2-000), (Fig. 1A), by: *i)* modifying purine moiety (MRTX; Fig. 1B), and *ii)* using a broader substitution pattern (MRT-XXX; Fig. 1C). Twenty-five analogues derived from R-roscovitine were first synthesised with only modification of the substituents (MRT2-XXX), representing chemical diversity, and then tested *in vitro* for their capacity to rescue the maturation of ER-retained class II ABCB4 variants, *i.e.* I490T, I541F and L556R, already described in previous studies^18–20^. For this first immunoblot screening, we used HEK293 cells (herein referred to as HEK cells), which allow high transfection rates and expression levels of the transgenes. Mature ABCB4 corresponds to the fully glycosylated protein (apparent MW ~ 160 kDa) present at the plasma membrane after complex glycosylation steps through the Golgi apparatus, as observed for the WT protein (ABCB4-WT), by opposition to incompletely glycosylated—and thus immature—forms with a higher electrophoretic mobility (apparent MW ~ 140 kDa) observed for class II ER-retained variants^21^. Immunoblot analyses indicated that some of these analogues partially rescue the maturation of the three class II ABCB4 variants (Fig. 2A, C, E). Quantification of these results revealed that MRT2-130 and MRT2-174 are able to partially but significantly rescue the maturation of the three ABCB4 variants, while MRT2-466 only rescues the maturation of ABCB4-I490T and -L556R (Fig. 2B, D, F). Cyclosporin A (CsA) was used as a positive control^22^ in our experiments. It is of note that some of the tested analogues lowered the expression of ABCB4 variants and/or induced cytotoxicity (apparent cell death) and were *de facto* excluded from further analyses; this includes MRT2-159, -165, -173, -207, -213, -233, -258, and -266 (Fig. 2A, C, E; quantification in Fig. 2B, D, F).

**Fig. 3:**
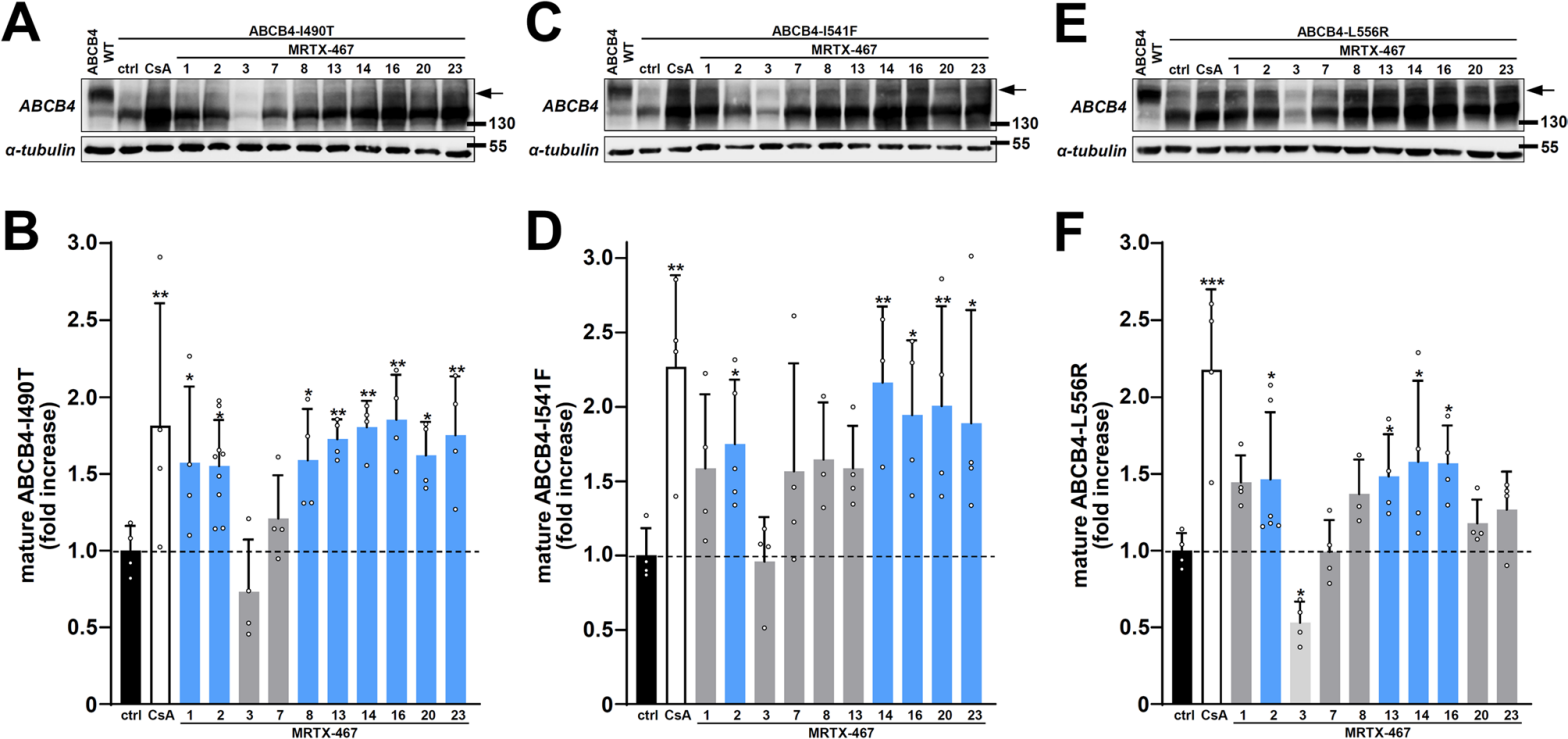
Maturation analysis of class II ABCB4 variants after treatment with roscovitine analogues with modified core. (**A**) HEK cells transiently expressing ABCB4-I490T were treated and analysed as in Fig. 2 after treatment with the indicated roscovitine analogues with modified core rings (see Fig. 1). (**B**) Densitometry analysis of (**A**), as in Fig. 2B. Means (± SD) of at least three independent experiments per condition are shown. Ctrl: control (vehicle); CsA: cyclosporin A. (**C**–**F**) Same as (**A**,**B**) with HEK cells expressing ABCB4-I541F (**C**,**D**) or ABCB4-L556R (**E**,**F**). (**A**,**C**,**E**) panels are representative of at least three independent experiments per condition. Full immunoblots are shown in Supplementary Fig. 5.

Then, we evaluated another series of ten roscovitine analogues with common substituents but modified number and/or position of nitrogen atoms in the purine core of the molecules (MRTX-467; see Fig. 1B). Among the tested analogues, maturation of: *i)* ABCB4-I490T was partially corrected by MRTX-467 analogues with cores #1, 2, 8, 13, 14, 16, 20, 23; *ii)* ABCB4-I541F was partially corrected by analogues MRTX-467 with cores #2, 14, 16, 20, 23; *iii)* ABCB4-L556R was partially corrected by analogues MRTX-467 with cores #2, 13, 14, 16 (Fig. 3A, C, E; quantification in Fig. 3B, D, F). MRT3-467 induced an important decrease of ABCB4 expression (Fig. 3A–F) and was therefore excluded from further analyses.

**Fig. 4:**
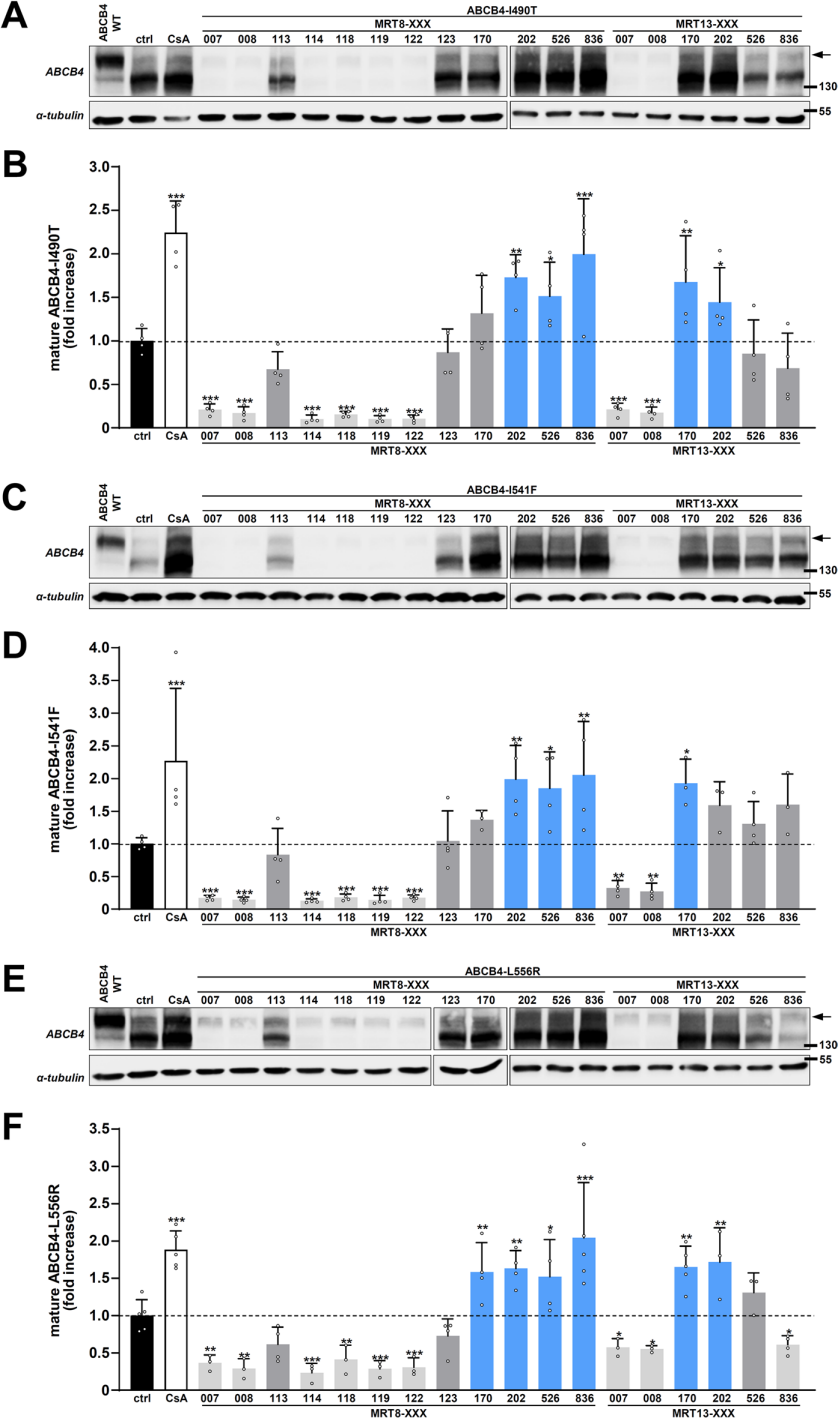
Maturation analysis of class II ABCB4 variants after treatment with a third series of roscovitine analogues. (**A**) HEK cells transiently expressing ABCB4-I490T were treated and analysed as in Fig. 2 after treatment with the indicated roscovitine analogues (see Fig. 1). (**B**) Densitometry analysis of (**A**), as in Fig. 2B. Means (± SD) of at least three independent experiments per condition are shown. Ctrl: control (vehicle); CsA: cyclosporin A. (**C**–**F**) Same as (**A**,**B**) with HEK cells expressing ABCB4-I541F (**C**,**D**) or ABCB4-L556R (**E**,**F**). (**A**,**C**,**E**) panels are representative of at least three independent experiments per condition. Full immunoblots are shown in Supplementary Fig. 6.

Finally, we selected a series of eighteen analogues based on MRT8-XXX and MRT13-XXX core rings, nine on each core rings (see Fig. 1B). Among these molecules, eight of them (MRT8-007, -008, -114, -118, -119- 122 and MRT13-007, -008) were cytotoxic (apparent cell death) and/or drastically reduced expression of ABCB4 variants (Fig. 4A, C, E; quantification in Fig. 4B, D, F) and were thus not considered for further analyses. However, MRT8-202, -526, -836 and MRT13-170 significantly rescued the maturation of the three class II ABCB4 variants, MRT13-202 rescued the maturation of I490T and L556R variants, while MRT8-170 only rescued ABCB4-L556R maturation (Fig. 4A, C, E; quantification in Fig. 4B, D, F).

Altogether, these results show that selected roscovitine analogues with a diversity of core rings and substituents constitute promising correctors for class II ABCB4 variants.

### Rescue of canalicular targeting of traffic-defective ABCB4 variants by selected roscovitine analogues

**Fig. 5:**
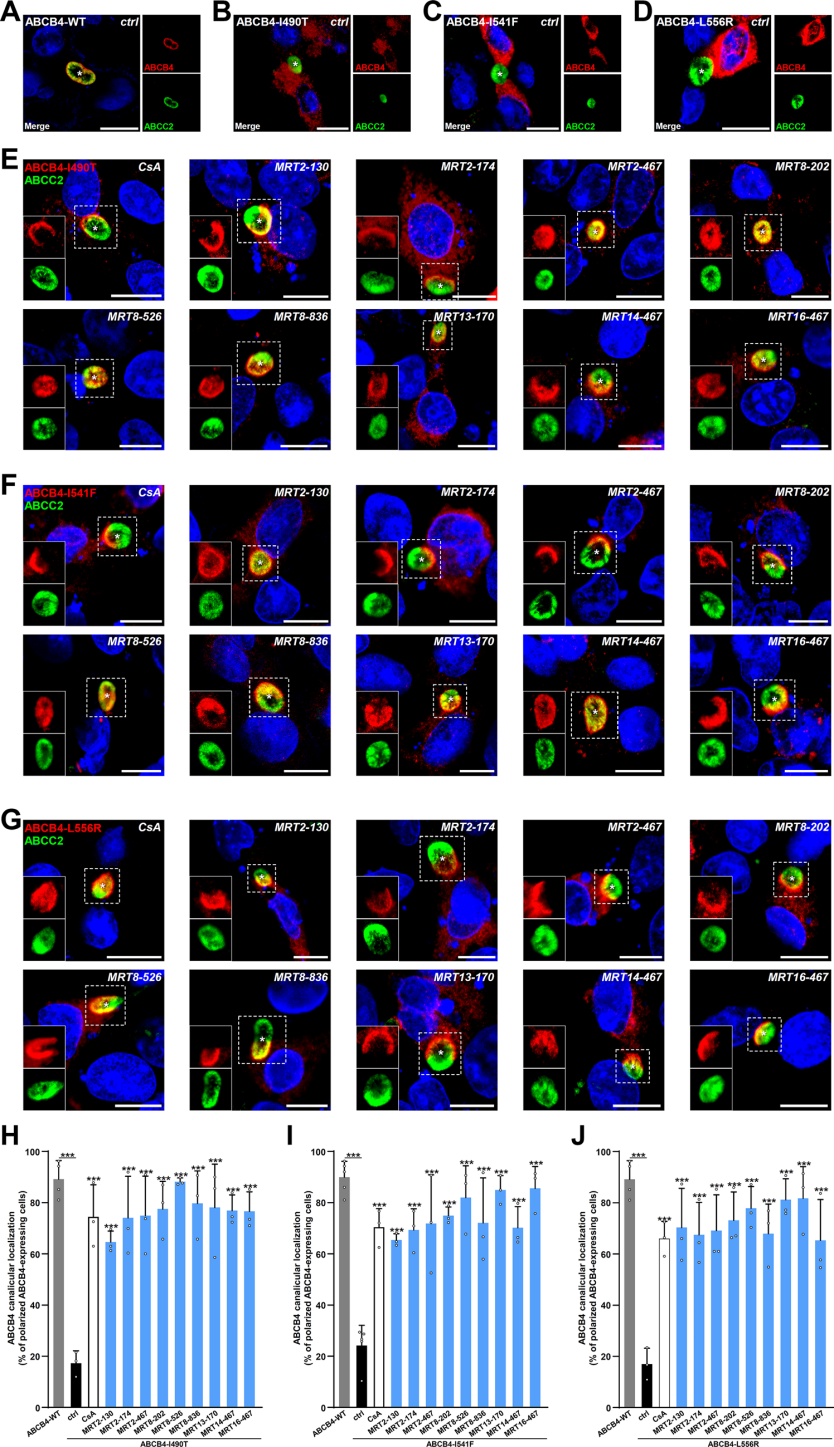
Selected roscovitine analogues partially correct the canalicular localisation of class II ABCB4 variants. (**A**–**D**) HepG2 cells transiently expressing ABCB4-WT (**A**), -I490T (**B**), -I541F (**C**) or L556R (**D**) were fixed and permeabilised after mock treatment (16 h with 1/1,000 DMSO). Cellular localisation of ABCB4 (red) and the canalicular marker ABCC2 (green) was then visualised by confocal microscopy after indirect immunofluorescence labelling. Nuclei shown in the merged images were labelled with Hoechst 33,342 (blue). (**E**–**G**) HepG2 cells transiently expressing ABCB4-I490T (**E**), -I541F (**F**) or L556R (**G**) were processed as in (**A**–**D**), except that cells were treated for 16 h with the indicated roscovitine analogues (10 µM) prior to fixation. Panels (**A**–**G**) are representative of at least three independent experiments per condition. White asterisks indicate bile canaliculi and the individual red (ABCB4) and green (ABCC2) of each merged image (delineated by dotted squares) are shown at the bottom left for each condition. Bars: 10 µm. (**H**–**J**) Quantification of (**E**–**G**), respectively. The canalicular localisation of class II ABCB4 variants (partial or complete colocalisation with ABCC2) was determined from > 200 cells from three independent experiments for each condition. Means (± SD) of three independent experiments are shown. Blue bars indicate statistical significance for the tested roscovitine analogues.

Based on the above screening of 53 roscovitine analogues and their capacity to partially rescue the maturation of the three investigated class II ABCB4 variants (Figs. 2, 3, 4), nine of them—all able to significantly rescue the maturation of the three class II ABCB4 variants studied—were selected for further analyses: MRT2-130, MRT2-174, MRT2-467, MRT8-202, MRT8-526, MRT8-836, MRT13-170, MRT14-467, MRT16-467 (Supplementary Fig. 1). First, we analysed their potential cytotoxicity in dose–response MTT release experiments, still in HEK cells: they were not significantly cytotoxic for all analogues at 10 µM (Supplementary Fig. 2). However, at higher doses (50 and 100 µM), some of these compounds become significantly cytotoxic, as observed in our previous study^18^, albeit in different ranges (Supplementary Fig. 2). In order to confirm our maturation analyses in experimental conditions closer to the pathophysiological situation, we performed indirect immunofluorescence in HepG2/C3A cells (herein referred to as HepG2 cells), a hepatocellular-derived cell line able to form pseudo-canaliculi in culture. By opposition to ABCB4-WT which mostly colocalises with the canalicular marker ABCC2 (Fig. 5A), the three class II ABCB4 variants are intracellularly-retained and do not colocalise with ABCC2 (Fig. 5B–D), as already reported^18–20^. In addition to CsA, used here as a positive control, the nine selected roscovitine analogues were all able to rescue, at least partially, the canalicular localisation of the three class II ABCB4 variants, ABCB4-I490T, -I541F and -L556R, as exemplified by confocal microscopy images (Fig. 5E–G). Quantification of these results confirmed that the nine roscovitine analogues allow the detection of ABCB4 variants at the canalicular membrane in 60 to 80% of ABCB4-expressing cells forming canaliculi (levels comparable to CsA-treated cells) while this percentage is ~ 20% before treatment for the three variants (Fig. 5H–J). These immunolocalisation analyses confirmed our previous maturation tests (see Figs. 2, 3, 4), indicating that the nine selected roscovitine analogues are potent ABCB4 corrector candidates.

### Effect of selected roscovitine analogues on the secretory function of wild type ABCB4 and class II variants

**Fig. 6:**
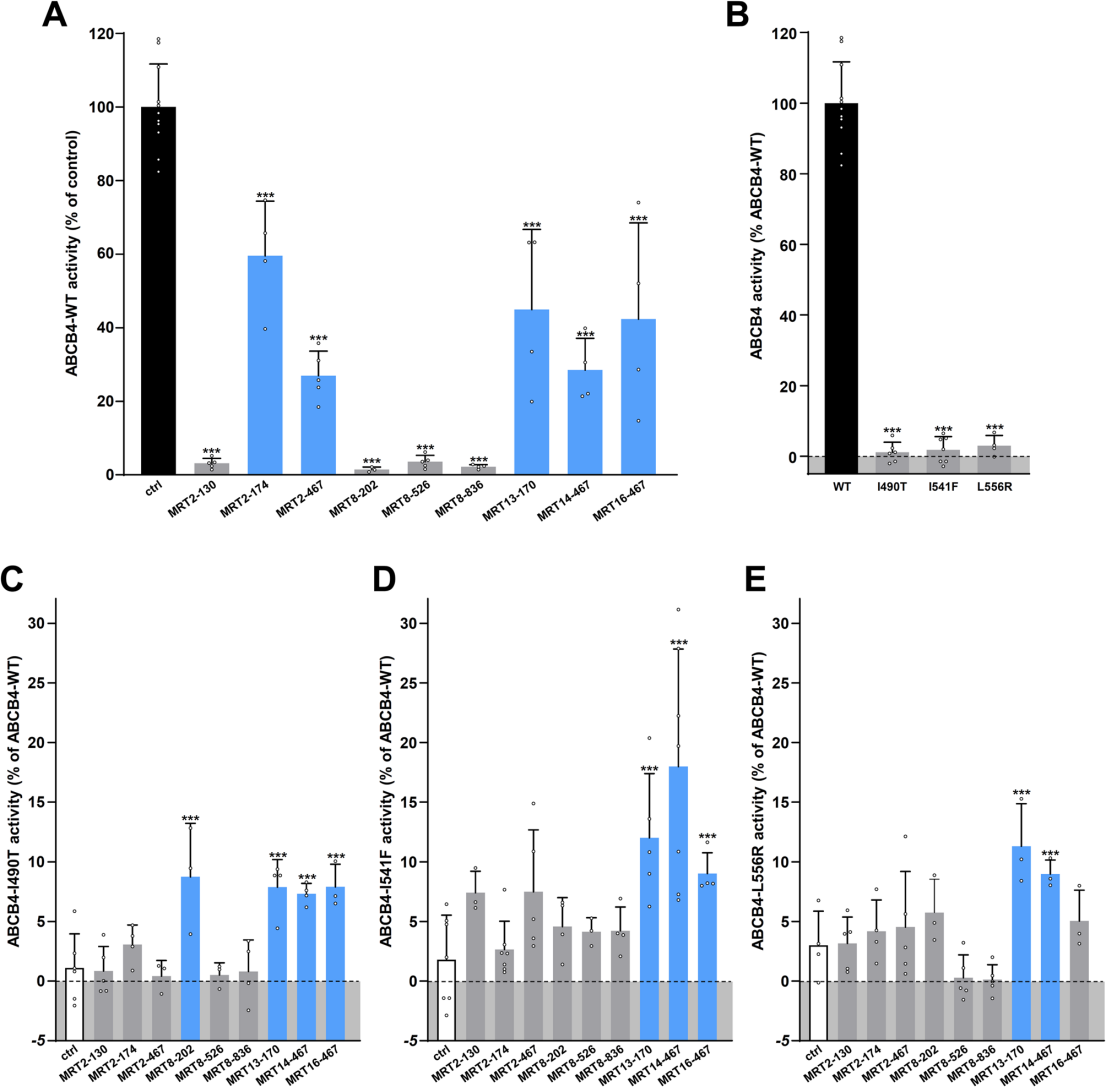
Effect of selected roscovitine analogues on the PC secretory function of ABCB4-WT and class II variants. (**A**–**E**) HEK cells expressing ABCB4-WT or variants were treated with vehicle (ctrl, DMSO) or 10 µM of the indicated roscovitine analogues. ABCB4-mediated PC secretion was measured, normalised to ABCB4 expression levels, and expressed as a percentage of the activity of vehicle-treated cells expressing ABCB4-WT for ABCB4-WT after treatment (**A**), ABCB4-WT versus variants without treatment (**B**), or I490T (**C**), I541F (**D**) and L556 variants (**E**) after treatment with roscovitine analogues. For (**B**–**E**), the grey areas indicate values below zero with the dashed lines representing 0% activity. Means (± SD) of at least three independent experiments performed in triplicate for each tested condition are shown. Blue bars indicate compounds allowing a significant remaining ABCB4-WT function (**A**), or statistical significance (**C**–**E**).

After selecting ABCB4 corrector candidates, it is of utmost importance to evaluate the capacity of traffic-rescued transporters to secrete PC into the extracellular environment, *i.e.* into bile canaliculus for hepatocytes. Indeed, we have previously shown that potent ABCB4 traffic correctors can only poorly—or even not at all—rescue PC secretion in drug-treated cells and moreover that such molecules can be potent ABCB4-WT inhibitors^18–20^. Measurement of ABCB4-mediated PC secretion was performed in HEK cells as published^18–20^ since polarized hepatocyte-derived cells are not suitable for such approaches (see Discussion). In HEK cells expressing ABCB4-WT, MRT2-130, MRT8-202, MRT8-526 and MRT8-836 were very strong inhibitors of ABCB4-WT, with < 5% remaining PC secretion activity after drug treatment (Fig. 6A). These four molecules were also not able to significantly rescue the activity of the three ABCB4 variants (Fig. 6B; activity of ABCB4-WT *vs* variants without drug treatment), with the exception of MRT8-202 on ABCB4-I490T (Fig. 6C–F). MRT2-174 and MRT2-467 were not strong ABCB4-WT inhibitors, with 59.56 ± 14.87% and 26.97 ± 6.67% of remaining PC secretion function, respectively (Fig. 6A), but these two analogues could not significantly rescue the function of the three ABCB4 variants (Fig. 6C–F). Finally, and more interestingly, the last three molecules, MRT13-170, MRT14-467 and MRT16-467, were not strong inhibitors of ABCB4-WT activity, with 45.0 ± 21.8%, 28.5 ± 8.7% and 42.4 ± 26.1% of remaining PC secretion function, respectively (Fig. 6A), and allow a partial but significant functional rescue of the three variants, except for MRT16-467 with ABCB4-L556R. We would like to emphasise that, due to the normalisation process of these functional assays (see Materials and Methods section), some close-to-zero values can be negative, as observed here in conditions with very low PC secretion activity (Fig. 6C–F). Altogether, our results indicate that selected roscovitine analogues with specific substituents and cores (see Discussion section) may be considered as promising correctors leading to a partial but significant rescue of PC secretory activity of class II ABCB4 variants.

### Insights into the potential structural impact of ABCB4 variants

**Fig. 7:**
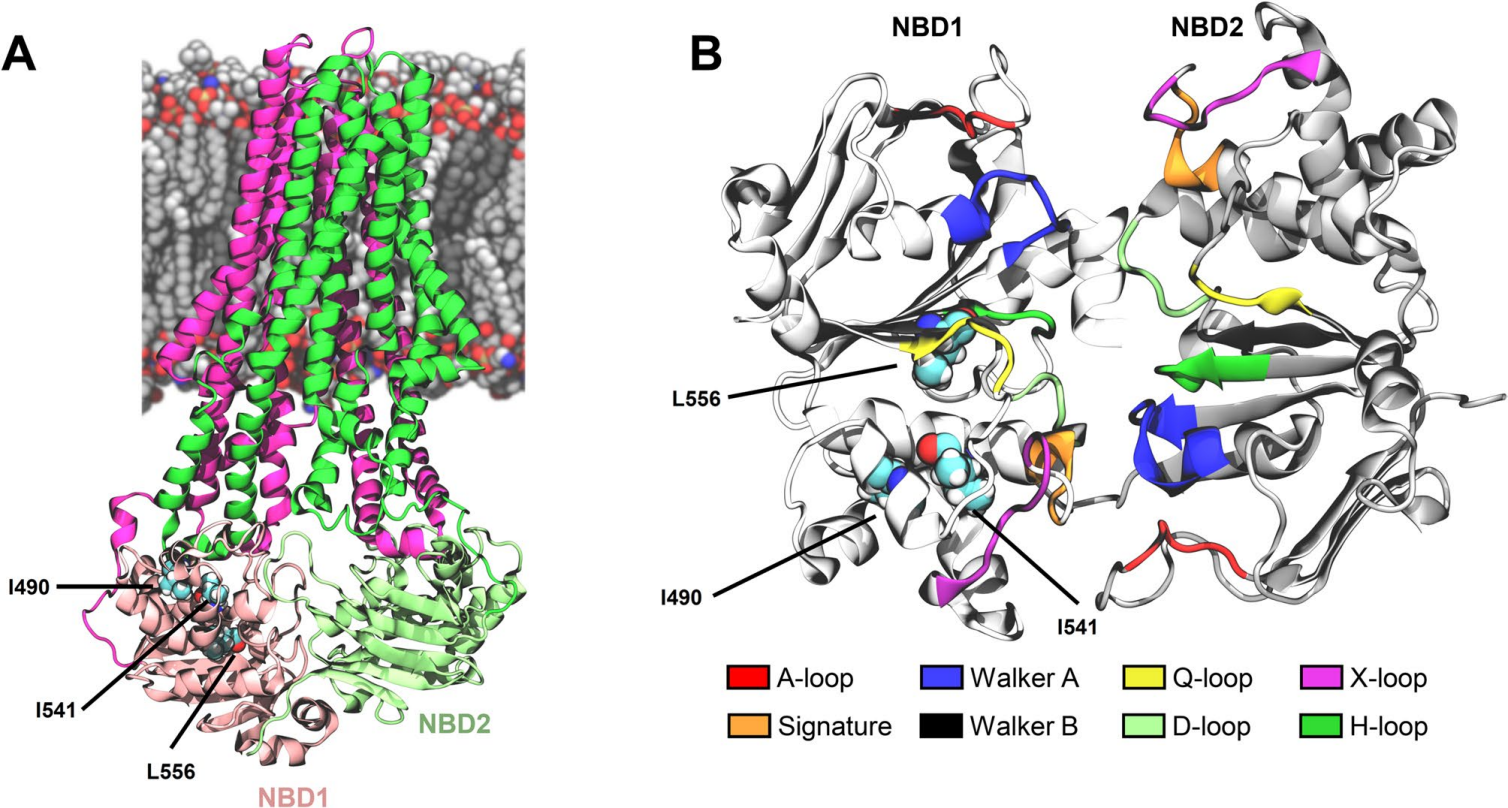
Structural overview of ABCB4 and location of disease-associated variants. (**A**) Representative snapshot of the wild type (WT) inward-facing (IF) ABCB4 model embedded in a lipid bilayer. Transmembrane helices (TMHs) 1–6 and 7–12, nucleotide-binding domain 1 (NBD1), and nucleotide-binding domain 2 (NBD2) are coloured purple, green, pink, and lime, respectively. (**B**) Close-up view of the NBDs showing the location of the three studied residues, I490, I541, and L556 with spheres representing atoms of oxygen (red), nitrogen (dark blue), carbon (light blue) and hydrogen (white). Conserved motifs involved in ATP binding and hydrolysis are highlighted, including the A-loop, signature motif, Walker A and Walker B motifs, and the Q-, X-, D-, and H-loops.

To decipher binding modes of selected roscovitine analogues, we generated putative structural models of ABCB4 variants by alchemical transformations. The I490T, I541F, and L556R variants are all located within the nucleotide-binding domain 1 (NBD1; Fig. 7A,B). Specifically, Ile490 lies between the Q- and X-loops, Ile541 is positioned before the Walker B motif, and Leu556 is located in the Walker B, which is responsible for nucleophilic attack during ATP hydrolysis step. It is important to note that the present computational approach does not account for large-scale unfolding of protein domains or global destabilisation caused by missense mutations. However, given that functional rescue of ABCB4 has been observed in the presence of correctors, we here hypothesised that the I490T, I541F, and L556R mutations do not substantially disrupt the secondary nor tertiary structure of ABCB4.

**Fig. 8:**
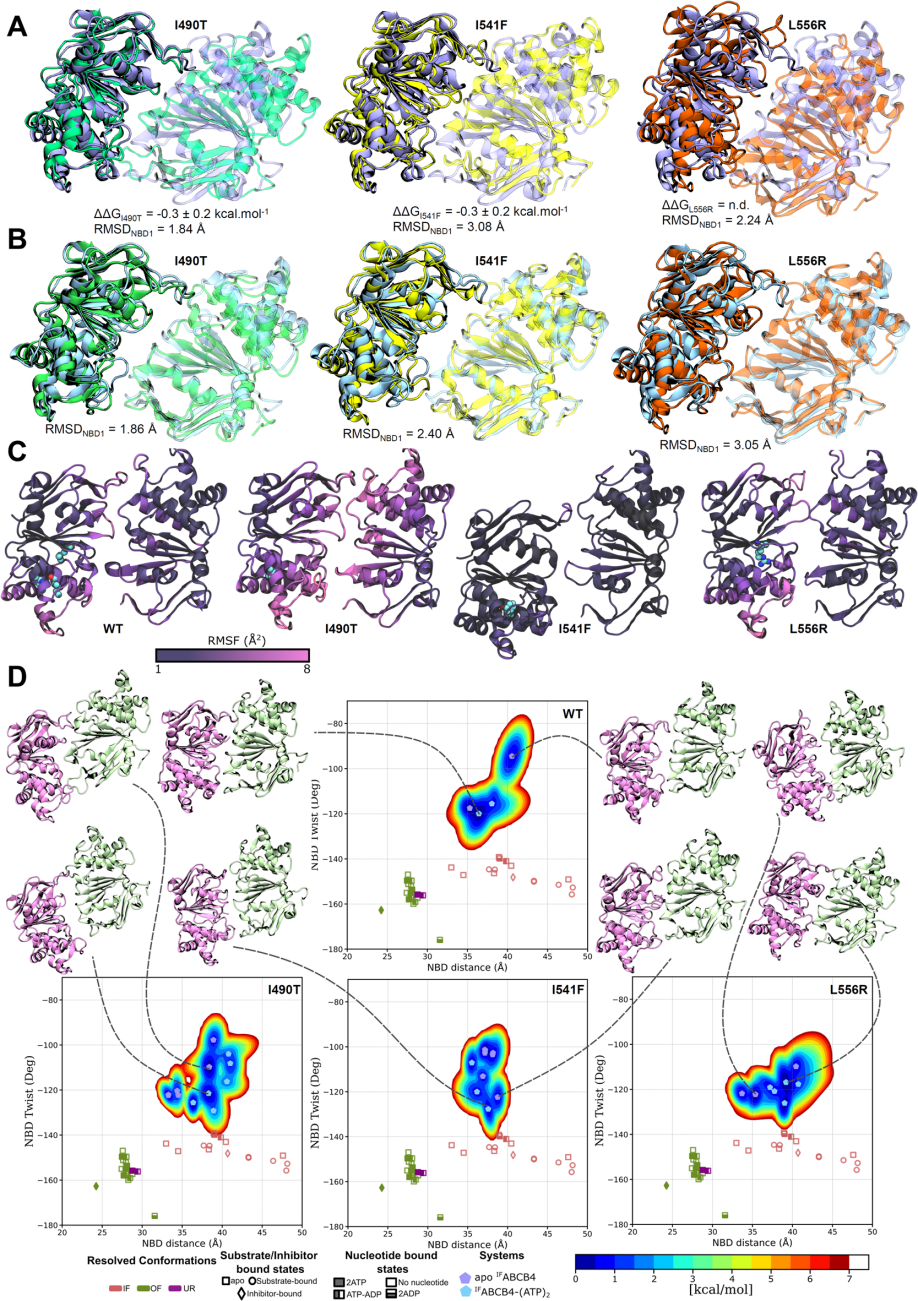
Molecular modelling of ABCB4-WT and class II variants. (**A**,**B**) Structural superimposition (quantified by NBD1 backbone root-mean squared deviation RMSD ± SD, n_simulations_ = 4) of NBD dimers of apo ^IF^ABCB4 (**A**) and ^IF^ABCB4-(ATP)_2_ (**B**). For the former, alchemical local free energy differences are shown. ABCB4-WT and I490T, I541F, L556R variants are coloured blue, green, yellow and orange, respectively. NBD1 and NBD2 are shown opaque and transparent, respectively. (**C**) Backbone flexibility of WT, I490T, I541F and L556R apo-^IF^ABCB4 models depicted according to backbone root-mean squared fluctuations (RMSF), with n_simulations_ = 4. Spheres represents the atoms of the residues of interest (colour-coded as in Fig. 7B), namely I490, I541 and L556 for ABCB4-WT structure, mutated T490 for I490T mutant, F541 for I541F mutant and R556 for L556R mutant. (**D**) InfleCS free energy-based conformational landscape calculated over all simulations (n = 4) according to the NBD twist and NBD distance parameters. Representative snapshots are shown for each main cluster. NBD1 and NBD2 are coloured in green and pink, respectively. IF: inward-facing; OF: outward-facing; UR: unlock-returned.

For I490T, the substitution of isoleucine with threonine appears unlikely to cause significant steric hindrance or structural deformation in the surrounding environment. Additionally, the calculated free energy differences suggested that I490T and I541F variants does not lead to destabilisation nor overstabilisation of NBD1, suggesting no major structural perturbation (Fig. 8A; ΔΔG is − 0.3 ± 0.2 kcal.mol^-1^ for both I490T and I541F variants). The free energy difference for L556R could not be reliably determined due to the introduction of a charge change owing to electrostatic decoupling associated with large Coulombic energy variations. For the sake of comparison, the relative free energy differences were also estimated using the deep-learning-based method available on the DDMut webserver^23^, for which the mutations were systematically associated with similar small destabilization, ΔΔG being -2.6 ± 0.2, -2.9 ± 0.2 and -2.2 ± 0.6 kcal.mol^-1^ for I490T, I541F and L556R, respectively (Supplementary Table 1). To further assess structural deviations, root-mean-square deviations (RMSD) of NBD1 were calculated relative to the WT model by superimposing NBD dimer, yielding values of 1.84 Å for I490T, 3.08 Å for I541F, and 2.24 Å for L556R. These results suggest that the mutations affect NBD1 structure and NBD dimer arrangement, likely due to altered interactions with NBD2. Notably, similar structural deformations in NBD1 were also observed in the inward-facing (IF) ^IF^ABCB4–(ATP)_2_ bound state (Fig. 8B; RMSDs being respectively 1.86 Å, 2.40 Å and 3.05 Å for I490T, I541F and L556R). Although molecular simulations indicate some mutation-induced modifications of NBD1, the domain’s stability appears to be preserved, particularly for the I490T and I541F variants. The overall secondary structure of NBD1 remains conserved (Fig. 8A,B), though its spatial arrangement relative to NBD2 appears slightly shifted. We further explored the dynamics of the NBD dimer during molecular dynamics (MD) simulations by calculating the root-mean-square fluctuations (RMSF), which reflect the local flexibility of residues (Fig. 8C). Interestingly, the three ABCB4 variants exhibited distinct dynamic behaviours compared to the WT protein. As compared with WT RMSD profile, the I490T variant showed a marked increase in flexibility across the entire NBD dimer, particularly at the dimer interface, suggesting potential destabilisation or increased conformational sampling of the NBD dimer. In contrast, the I541F variant displayed globally reduced flexibility, with RMSF values lower than those of WT, possibly indicating a more rigid NBD dimer. Finally, the L556R variant demonstrated flexibility levels comparable to WT, suggesting that the local dynamics of the NBD dimer are preserved.

To further investigate the structural dynamics of ABCB4, we also analysed NBD-NBD distance and the NBD twist (Fig. 8D), two key conformational parameters of ABC transporters^24^. Both WT and variant ABCB4 models sampled similar regions of conformational space, with NBD distances varying between approximately 30–45 Å and NBD twist angles ranging from -130° to -90°. However, the corresponding free energy landscapes differed notably, particularly with respect to the conformational transitions between apo and ATP-bound states. In the WT protein, the apo and ATP-bound conformations remained at least partially distinct, with a subset of representative structures exhibiting a conformation consistent with the canonical antiparallel NBD dimer observed in functional transporters. ATP binding in ABCB4-WT slightly reduced the NBD distance and twist angle, promoting a more compact and dimerised configuration. In contrast, the ABCB4 variants demonstrated more fragmented conformational landscapes, characterised by an increased number of local minima and putative metastable states. Notably, in the I490T and L556R variants, ATP binding did not consistently induce the expected NBD compaction; in some cases, ATP-bound conformations displayed even larger NBD distances than their apo counterparts. This observation, likely linked to increased backbone flexibility, suggests a compromised ability of the variant proteins to form or maintain a stable NBD dimer. These findings imply that the conformational coupling between ATP binding and NBD dimerisation is weakened in ABCB4 variants.

### Potential binding between roscovitine-based correctors and ABCB4 models

**Fig. 9:**
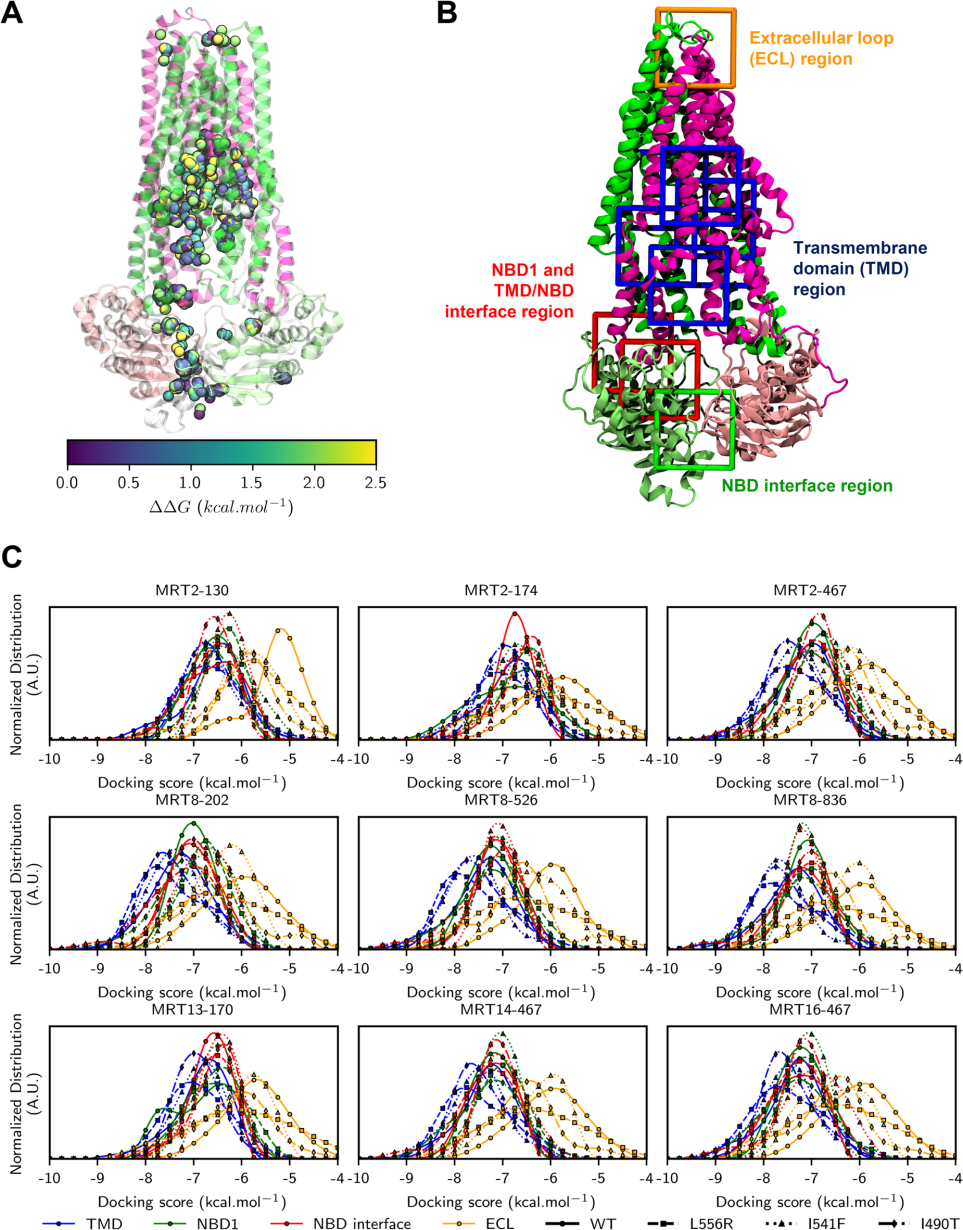
Molecular docking of selected roscovitine analogues with ABCB4-WT and class II variants. (**A**) The selected centres of mass for MRT2-130, MRT2-174, MRT2-467, MRT8-202, MRT8-526, MRT8-836, MRT13-170, MRT14-467, MRT16-467 obtained from brute-force blind molecular docking for which best poses were selected with a docking score of 2.5 kcal.mol^-1^. (**B**) Preferential binding regions obtained from blind molecular docking that was also used for refined flexible molecular docking. TMD, NBD1, NBD dimer interface and ECL regions were respectively sampled from 6, 2, 1 and 1 volume search spaces (coloured blue, red, green and orange, respectively). (**C**) Normalised docking score distribution calculated from refined flexible ensemble docking conducted on WT, I490T, I541F and L556R ^IF^ABCB4 models. Distributions are shown according to binding region (coloured blue, red, green and orange, respectively for TMD, NBD1, NBD dimer interface and ECL) and ABCB4 models.

Based on the hypothesis that correctors may restore ER-retained ABCB4 variants through direct interactions as suggested for CFTR caftors^25^, we evaluated their potential binding affinities using molecular docking calculations. Although roscovitine analogues possess a purine-like core similar to ATP, we adopted a blind, brute-force molecular docking strategy in the absence of prior knowledge regarding their binding sites (Supplementary Fig. 7). The entire protein structure was exhaustively sampled by dividing it into sub-volumes, resulting in the generation of over 50 million molecular poses. Despite this extensive sampling, the predicted binding sites were concentrated in a limited number of regions (Fig. 9A). Specifically, only four major binding regions were consistently identified: the extracellular loop (ECL), the transmembrane domain (TMD) including the substrate binding site, NBD1, and the NBD interface. Notably, and in contrast to previous investigations with other molecules^19,20^, NBD2 did not emerge as a significant direct binding site for any of the roscovitine analogues, with the exception of few poses (Fig. 9B).

Refined flexible molecular docking calculations were subsequently performed to refine the potential binding modes of selected roscovitine analogues within the previously identified regions. Overall, all selected analogues demonstrated the capacity to directly interact with both ABCB4-WT and variants, with predicted binding affinities ranging from -10.5 to -4.5 kcal·mol⁻^1^ (Fig. 9C). These results indicated that roscovitine analogues tend to bind either within the TMD or NBD1 as pictured by distribution of energy score (Fig. 9C). Interestingly, binding modes of correctors in NBD1 region showed potential interactions at the NBD1-TMD interface as already proposed for ivacaftor with CFTR class III variant^25^. The ECL region is the less favourable region for roscovitine analogues binding (Fig. 9C). Unfortunately, molecular docking calculations does not suggest differential binding of roscovitine analogues between ABCB4-WT and class II variants. This might be explained by the poor proposed structural impact arising from mutations (Fig. 8). Contact analyses were conducted to identify specific residues involved in roscovitine analogues; but no significant pattern was observed according to either ABCB4 variants or roscovitine analogues (Supplementary Fig. 8). Indeed, no specific binding mode was identified to rank the nine selected roscovitine analogues owing to the inherent chemical accuracy of molecular docking. Interestingly, MRT2-XXX roscovitine analogues (except for MRT2-467) showed almost no difference in binding either TMD or NBD regions, while MRT13-170, MRT14-467, MRT16-467, MRT8-202, MRT8-526, and MRT8-836 slightly bind preferentially TMD rather than NBD1. This might be explained by the core pattern which is less planar for MRT8, MRT13, MRT14 and MRT16 than MRT2. However, MRT2-467 exhibit similar profile as non-MRT2, likely owing to bulkier R2 moiety than MRT2-130 and MRT2-174.

We would like to emphasise that the present docking protocol assumed that the overall secondary and tertiary structures of the NBDs, particularly NBD1, are preserved despite the presence of mutations; an assumption that may not fully hold, especially in the absence of experimental confirmation of protein folding. Nonetheless, these results support the hypothesis that roscovitine analogues may exert their corrective effect through direct interaction with ABCB4, primarily targeting sites within the TMD and NBD1.

## Discussion

Genetic variations of ABCB4 are associated with rare cholestatic liver diseases, the most severe form of these diseases being PFIC3^8,12^. Class II variants affect the intracellular traffic of the transporter, leading to impaired PC secretion^13,14^. We have previously identified several ABCB4 correctors, including roscovitine analogues, ABCC7/CFTR correctors and hits from a high-content screening, but their capacity to restore PC secretion was limited, which was correlated with their potent inhibitory effect on ABCB4-WT function^18–20^. In the present study, we pursued our efforts towards roscovitine analogues by testing a larger number of molecules than in our previous work^18^, more specifically by introducing a greater variability of the roscovitine purine core and the substitution pattern. At 10 µM, roscovitine (MRT2-000) did not provide significant rescuing effect by opposition to previous studies in which it was used at 100 µM^17,18^, while some structural analogues did, suggesting that they are more potent correctors than the parent molecule itself. Among the 53 tested molecules, nine analogues were preselected, based on their capacity to rescue protein maturation and canalicular targeting of class II ABCB4 variants; and two of them (MRT13-170 and MRT14-467) significantly increased ABCB4-mediated PC secretion for the three variants with moderate inhibitory effects on ABCB4-WT function.

We also investigated the impact of the ABCB4 missense variations on the overall structure of the transporter using an alchemical free energy calculation approach. Although this strategy has proven its effectiveness^26^, it did not reveal significant structural alterations in any of the three ABCB4 variants, nor notable stabilization or destabilization effects for the I490T and I541F variants. Due to the limitations of alchemical side-chain transformations, the free energy difference for the L556R variant was not assessed, as this mutation involves a change from a neutral to a cationic residue. We additionally evaluated the free energy difference using a deep learning-based method (DDMut)^23^, which indicated minimal destabilization upon single-point mutation (approximately -2.5 kcal.mol^-1^). Considering the chemical accuracies of the two approaches, ranging from 1.0 to 1.5 kcal.mol^-1^ for alchemical transformations^26^ and 1.78 kcal.mol⁻^1^ for DDMut^23^, our results suggest that the I490T, I541F, and L556R mutations are unlikely to induce major local structural disruptions. Two non-mutually exclusive explanations can be proposed: *i)* these variants indeed do not cause substantial structural changes, and their trafficking defect may stem from other mechanisms; or *ii)* the computational methods used are not suited to detect structural impairments arising during protein translation, which may lead to NBD1 misfolding. The first hypothesis is supported by the observation that class II ABCB4 variants can retain partial activity after pharmacological rescue of plasma membrane targeting as previously reported for other compounds^18,20^, indicating that their ability to bind and hydrolyse ATP remains preserved, something unlikely if dramatic structural rearrangements had occurred. It is thus tempting to hypothesize that minor structural or sequence alterations may modulate the dynamics of ABCB4 NBD, as suggested by RMSF analyses and local free energy surface differences obtained from MD simulations. Similar mechanisms have been described for ABCC7/CFTR, where a single-point mutation in NBD1 alters its dynamics and subsequently disrupts interactions with other protein domains, leading to enhanced clearance by the cellular quality control system^27^. A comparable mechanism could occur for ER-retained ABCB4 variants, where NBD1 mutations might perturb dynamic interactions with molecular chaperones and/or ER export machinery components such as SEC24 proteins, part of the COPII complex^28,29^, as shown for ABCC7/CFTR-F508del^27,30^. Further structural studies are therefore needed to explore this hypothesis in greater detail.

In this study, we used cell models to characterize the effect of roscovitine analogues on the expression, traffic and function of class II ABCB4 variants. While unpolarized HEK cells are suitable for rapid screening of compounds (immunoblots and cytotoxicity assays) thanks to high transfection rates and expression levels of transgenes, we then shifted to hepatocyte-derived HepG2 cells, which is a more physiologically relevant model and allows better subcellular localisation analyses since these cells are polarized and form pseudo-canaliculi^31^. However, HepG2 cells are not adapted for further functional PC secretion assays, mostly because transfection rates are not optimal in this cell line (< 50%) and, more importantly, because the closed environment of the pseudo-canaliculi formed by the apical/canalicular membrane of these cells makes it very challenging to specifically collect secreted PC. Thus, we used HEK cells again for functional assays, as we have done in the frame of our previous studies^18–20^. Although not perfectly adapted to the physiological situation, this *in vitro* human system provides clues for further functional analyses using more physiological strategies, the development of which still being needed. Immunolocalisation analyses in HepG2 cells also showed a partial canalicular localisation (with a concomitant partial intracellular staining) of ABCB4 variants after treatment with roscovitine analogues. This may be explained by a weaker efficiency of some molecules to rescue the canalicular localisation of the variants, or more likely by the fact that the transient expression system used here led to a greater cell-to-cell variability of the transgene expression, *i.e.* the canalicular rescue may appear as partial due to an important intracellular background in an overexpression context. In the same line, it is also important to note that some roscovitine analogues (*e.g.*, MRT2-130, MRT2-174, MRT8-202, MRT8-526, MRT8-836, MRT13-202, MRT16-467, MRT20-467) induce a general overexpression of ABCB4 (both mature and immature forms), as also observed with the positive control CsA. The molecular mechanisms behind this overall overexpression upon drug treatment remain unknown but this may have an impact on the unfolding protein response^32^, an aspect that paves the way for further investigations.

Roscovitine is a trisubstituted purine^16^ and its structural analogues share this general feature for which molecular planarity may be slightly modulated. This may explain the inhibitory effect of planar roscovitine analogues, at different scales, on ABCB4-WT activity and/or the heterogeneity of transporter-mediated PC secretion of cells expressing ABCB4 variants. Due to the purine-like structure of roscovitine and its analogues, they may compete with ATP at ABCB4 nucleotide-binding sites, precluding its binding and hydrolysis by the transporter^20,33^. Indeed, such a hypothesis is supported by our *in silico* docking calculations indicating that roscovitine analogues may bind the ATP-binding site 1, as similarly observed with ABCC7/CFTR correctors^19^ and hits from high-content screening^20^. However, two of the roscovitine analogues identified in the present study (MRT13-170 and MRT14-467) allow a partial rescue of ABCB4-mediated PC secretion for the three tested class II variants, with a limited inhibitory effect on ABCB4-WT function. Overall, chemical variations of roscovitine core have a limited impact on the effect of the different analogues while substitution pattern can more significantly affect cell viability, ABCB4 expression and/or the rescuing capacity of the molecules. Concerning R2 substituents, our results do not allow us to conclude about cytotoxic effects or their rescuing capacity. However, it is clearer that molecules bearing R2 substituents such as chloride, fluor, *N*-methyltetrahydrofuranamine are better correctors and/or less ABCB4-WT inhibitors than others that might affect binding mode owing to *e.g.*, H-bond interactions for substituent bearing hydroxyl moieties. For R6 substituents, it is interesting to note that the all nine selected molecules contain a bulky aromatic moiety, either *N*-methyl-phenylmethanamine or *N*-methyl-pyridinylmethanamine, while most molecules with toxic/ABCB4-expression-lowering effects contain even larger, more flexible and more charged R6 substituents, such as *N*-methyl-pyridinylphenylmethanamine or fluoro/chloro-indol-*N*-methylmethanamine. The variability of R9 substituents was not investigated since most of these moieties are pretty similar (isopropyl, isocyclodecanyl). This is in line with our previous observations showing that such modifications did not have a strong effect on the rescuing capacity of roscovitine analogues^18^. Altogether, we are convinced that these observations will guide further structure–activity relationship (SAR) studies, which will allow the design of a new generation of roscovitine analogues with better ABCB4 rescue/inhibition ratios, as performed with the ABCC7/CFTR corrector VX-809/lumacaftor, also inhibitor of the channel function^34^, which was further optimized towards improved molecules, namely VX-445/elexacaftor and VX-661/tezacaftor (for a review, see^35^).

Molecular docking analyses suggest that roscovitine analogues might bind within various regions of ABCB4, including the TMDs, the NBD1/TMD interface, or the inter-NBD interface. Although the limited chemical accuracy of docking methods prevents the reliable identification of a preferred binding site, considerations of structural accessibility may provide insights into the molecular mechanism underlying ABCB4 rescue. Given that immature ABCB4 proteins localized in the ER have their NBDs oriented towards the cytosol, NBD binding regions are likely more accessible than the inner cavity of the TMDs. This idea aligns with findings for ABCC7/CFTR, where stabilization of NBD1 was associated with enhanced rescue of the F508del mutant. Accordingly, roscovitine analogue binding within the NBD region could stabilize the NBD/TMD interface, preventing ABCB4 degradation by the cellular quality control system^36^. Such a mechanism could also explain the limited transport activity of rescued ABCB4, as roscovitine analogues might partially compete with ATP for binding. From a molecular design perspective, these observations may guide the development of next-generation analogues capable of binding NBD1 with moderate affinity, sufficient to stabilize the protein without interfering significantly with ATP binding. Ultimately, beyond computational docking predictions, these potential small-molecule interactions with ABCB4 should be validated experimentally using structural biology approaches such as cryogenic electron microscopy, as already demonstrated for purified ABCB4 complexed with Fab fragments or posaconazole^37^.

Concerning therapeutic perspectives of ABCB4 correctors, we would like to emphasise that the goal is not necessarily to reach a full restoration of function for ABCB4 variants. Indeed, reaching a threshold of PC secretion into bile would be sufficient to make patients better responders to combinatory therapies with UDCA or potentiators as VX-770/ivacaftor, the latter being shown to partially rescue the function of selected class III ABCB4 and ABCB11 variants^38–41^. We have proposed that ~ 7% of PC among total biliary lipids would be the threshold allowing a positive response of PFIC3 patients to UDCA^12^, compared with 19–24% in healthy individuals^9^, thus representing a 28–36% (6.9/24 to 6.9/19) threshold of ABCB4 activity to reach after pharmacological rescue. In addition, a cell therapy approach showed that a repopulation of 12% of wild type hepatocytes in *Abcb4*^*-/-*^ mouse cells would be sufficient to abrogate the liver pathology^42^, while a gene therapy strategy in *Abcb4*^*-/-*^ knock-out mice indicated that only 2–3% of Abcb4-WT expression would be sufficient to exert a therapeutic effect^43^. Finally, since restoring 5% of wild type ABCC7/CFTR mRNA levels would be enough to protect from severe cystic fibrosis, the threshold to limit evolution later in life being probably more comprised between 10 and 30%^44,45^. In this context, for class II ABCB4 variants with < 5–10% of residual activities (this study and^18–20^), our first goal would be to reach a 20–25% threshold of ABCB4-WT activity for the variants after pharmacological treatment. Reaching this threshold and lowering the inhibitory effects of pharmacochaperones on ABCB4-WT function will require further chemical optimisation of ABCB4 correctors, including on the foundation of the two promising roscovitine analogues identified in the present study, before considering their *in vivo* preclinical validation.

This low-scale screening of small molecules constitutes a new step towards the identification and characterisation of ABCB4 correctors, opening the path to medicinal chemistry programmes which will aim at the chemical optimisation of preselected hits, in combination with *in silico, in vitro* and *in vivo* experiments.

## Materials and Methods

### Chemical synthesis of roscovitine and analogues

The synthesis of roscovitine and each of its analogues is shown in Supplementary Fig. 3 and was performed as published^46–50^ and patented (World Intellectual Property Organization, WO2022061155; European Patent Organization, EP4268824). For data protection and patentability reasons, some compounds will be described and published later (Elie, J. et al*.*, 2025, submitted).

### Plasmids, cell culture, transfection and cell treatments

The pcDNA3 plasmid encoding ABCB4-WT has been previously described^21^. The three class II ABCB4 missense variants studied here (I541F, L556R and I490T) were also reported and characterised in other studies^7,13,18,20,21,51^. Human embryonic kidney (HEK-293, herein referred to as HEK; ATCC®-CRL-1573™) cells and human hepatocellular carcinoma HepG2/C3A (derivative of HepG2; herein referred to as HepG2; ATCC®-CRL-3581™) cells were obtained from ATCC (Manassas, VA, USA) and maintained at 37 °C in a humidified atmosphere containing 5% CO₂, following previously established protocols^19,20^. Transient transfections were carried out using FuGENE® HD (Promega, Charbonnières-les-Bains, France) for HEK cells and JetPrime® (Polyplus Transfection, Illkirch, France) for HepG2 cells, according to the manufacturers’ recommendations. Cells were seeded in 6-well plates and transfected with 1 µg (HEK) or 2 µg (HepG2) of plasmid DNA per well, 6 or 24 h post-seeding, respectively. Roscovitine analogues were solubilised in dimethylsulfoxide (DMSO) to prepare 1000X concentrated stock solutions (aliquoted and stored at -20 °C) and used at a 10 µM final concentration, which corresponds to gold standards in drug discovery programmes^52^. DMSO was used as a control vehicle at the same dilution (0.1% DMSO in all conditions). Twenty-four hours post-transfection (except for cytotoxicity assays without transfection, see Supplementary Methods), cells were treated during 16 h with these drugs before performing cytotoxicity assays, immunoanalyses and functional assays.

### Immunoanalyses and functional assays

Immunoblots were performed as previously described^18^. Briefly, cells were lysed in lysis buffer (1% Triton X-100 in 20 mM Tris–HCl pH 7.4, 150 mM NaCl, 1 mM EDTA), cell lysates were separated by 8% SDS-PAGE followed by immunoblotting using the following primary antibodies: mouse monoclonal anti-ABCB4 (clone P3II-26; Enzo Life Sciences, Villeurbanne, France) and anti-α-tubulin (clone 1E4C11; ProteinTech, Manchester, United Kingdom). Horseradish peroxidase-coupled secondary antibodies were from Merck/Sigma (Saint-Quentin-Fallavier, France). Proteins signals were detected by enhanced chemiluminescence using a Fusion Fx7 device (Vilber Lourmat, Collégien, France). Immunoblots were quantified in the linear range of detection using ImageJ 1.50i software (US National Institutes of Health). ABCB4 expression was normalised to α-tubulin expression levels.

Indirect immunofluorescence analyses were performed as reported^18^. Briefly, HepG2 cells grown on glass coverslips were fixed (ice-cold methanol) after transfection and drug treatment (see above) and processed using the following primary antibodies: mouse monoclonal anti-ABCB4 (clone P3II-26; IgG2b) and anti-ABCC2 (clone M2I-4; IgG1) from Enzo Life Sciences (Villeurbanne, France). The corresponding AlexaFluor™-conjugated isotype-specific secondary antibodies were from Thermo Fisher Scientific (Villebon-sur-Yvette, France). Nuclei were stained using Hoechst 33,342 (Thermo Fisher Scientific). Coverslips were mounted on glass slides using Mowiol 4.88 (Merck/Sigma). Immunofluorescence images were acquired using a confocal microscope (Eclipse TE-2000-Nikon-C2) equipped with a 60X 1.40 oil immersion objective. Among ABCB4-positive cells forming canaliculi, the percentage of cells expressing ABCB4 at the canalicular membrane was determined.

Measurement of ABCB4-mediated PC secretion was performed using a previously described fluoro-enzymatic assay^53^, with detailed information on data processing provided in our previous publication^19^. Briefly, media from transfected and drug-treated HEK cells were collected after 24 h and total lipids were extracted, evaporated and resuspended in PBS containing 0.1% Triton X-100. The fluorescence readout (λ_exc_: 320 nm; λ_em_: 404 nm) was measured with a multiplate fluorimeter (Varioskan LUX multiplate reader, Thermo Fisher Scientific). All measurements were performed in triplicate, normalised to ABCB4 expression levels and expressed as a percentage of ABCB4-WT secretion activity.

### Construction of *in silico* ABCB4 variant models by alchemical transformations

We recently proposed computational structural insights of ABCB4-WT adopting several conformation and bound states, apo ^IF^ABCB4, ^IF^ABCB4-(ATP)_2_, ^IF^ABCB4-PC, ^IF^ABCB4-PC-(ATP)_2_, ^cc^ABCB4-(ATP)_2_^54^. Four representative snapshots of apo ^IF^ABCB4, ^IF^ABCB4-(ATP)_2_ simulations were extracted using the free energy-based Inflection core state Clustering (InfleCS) method using the so-called ABC parameters^24,55^ as structural descriptors yielding a total of eight structures. It must be stressed that these models were natively embedded in asymmetric plasma membrane-like lipid bilayer. ER-retained ABCB4 variants (namely I490T, I541F, and L556R) were created by alchemical transformations using eight λ-windows at 0, 0.02544, 0.12923, 0.29707, 0.5, 0.70292, 0.87076, 0.97455, and 1. We here hypothesised that I490T, I541F and L556R mutations were not associated with a substantial misfolding of NBD1 structures. Therefore, alchemical transformations were conducted on the amino acid side chains, keeping WT backbone atoms. Alchemical transformations were conducted using softcore potential by smoothly switching off (on) both electrostatic and Lennard–Jones potential of WT (and variant) atoms. It is important to note that for the L556R variant, one sodium cation was also included in the softcore region to maintain system neutrality. For each window, systems were first minimised and equilibrated as described below. Importantly, the final structure of each λ_i_-window after equilibration was used as starting structure for the following λ_i+1_-window. Then, 100 ns production simulations were performed. Free energy differences were calculated by means of thermodynamic integration. Three alchemical replicas were carried per variant. For the sake of comparison, free energy differences between ABCB4-WT and single-point mutants I490T, I541F and L556R were also assessed by using the deep-learning based method available on the DDMUT webserver^23^. Briefly, DDMut assessed free energy differences by considering changes in the local environment between WT and single-point mutant by means of graph-based representations. Therefore, such a method also considers that single-point mutation does not change the local secondary structure. This has been achieved by using seven representative snapshots obtained from apo ^IF^ABCB4, ^IF^ABCB4-(ATP)_2_ simulations^54^. Importantly, DDMut was developed with a focus on soluble proteins, as opposed to ABCB4. However, since I490T, I541F and L556R mutations are located in NBD1, we here assumed the relevance of DDMut to assess free energy differences.

Since alchemical transformations were conducted in plasma membrane-like lipid bilayers, we embedded ABCB4 variant structures obtained after MD production calculated at λ = 0 in ER-like lipid bilayer membrane. Two lipid bilayer models were considered: *i) *the “simplistic” membrane, composed of 1-palmitoyl-2-oleyl-sn-glycero-3-phosphatidylcholine (POPC, 60%), 1-palmitoyl-2-oleyl-sn-glycero-3-phosphatidylethanolamine (POPE, 30%), Cholesterol (Chol, 10%); the “complex” membrane, made of six lipids, namely 1,2-dioleyl-sn-glycero-3-phosphatidylcholine (DOPC, 25%), POPC (35%), 1,2-dioleyl-sn-glycero-3-phosphatidylethanolamine (DOPE, 25%), Chol (10%), 1,2-dioleyl-sn-glycero-3-phospho-L-serine (DOPS, 3%), 1,2-dioleyl-sn-glycero-3-phosphatidic acid (DOPA, 2%). Both membranes have a symmetric composition as the membrane of the ER, by opposition to the plasma membrane. Results shown in the present manuscript are those using the complex membrane.

### Molecular dynamics setup

MD simulations were conducted with apo-^IF^ABCB4, ^IF^ABCB4-(ATP)_2_ models embedded in lipid bilayers, in four different replicas using the CPU and GPU-version of Amber22 pmemd code^56^. ^IF^ABCB4-(ATP)_2_ structures were obtained by docking ATP and Mg^2+^ molecules to the apo structures. FF14SB^57^, lipid21^58^, TIP3P^59^, and DNA.OL15-based^60^ forcefields were used to model amino acids, lipids, water, and ATP molecules, respectively. Mg^2+^ ions and NaCl were modelled using the mono and divalent parameters described in TIP3P water forcefield^59^. A cutoff of 10 Å was applied to both Coulomb and van der Waals interactions, while long-range electrostatics were treated using the particle mesh Ewald (PME) method^61^. In the MD simulations, first, minimisations using energy steepest gradient were conducted as follows: *i)* minimisation of water O-atoms for 20,000 steps, *ii)* all bonds involving H-atoms for 20,000 steps, *iii)* water molecules and counterions for 50,000 steps, and *iv)* the whole system for 50,000 steps. For each model, system thermalisations were performed using Langevin thermostat in two steps. First, water molecules were thermalised from 0 to 100 K for 50 ps in the NVT ensemble with 0.5 fs as integration time. Then, the whole system was heated up from 100 to 310 K for 500 ps in the NPT ensemble using a 2 fs integration time, and restraining bonds involving H-atoms with the SHAKE algorithm. Pressure and box equilibrations were then achieved by performing short MD simulations for 5 ns with a integration time set at 2 fs in the semi-isotropic NPT ensemble for which Berendsen barostat was employed^62^. Finally, for each model, MD productions of 2 µs were performed using 2 fs as integration time step, in the NPT ensemble condition with semi-isotropic scaling. Langevin dynamics thermostat with 1.0 ps^-1^ collision frequency was used to maintain the temperature. The overall pressure was maintained at 1 bar with semi-isotropic pressure scaling using Berendsen barostat. Noteworthy, ATP-bound models were carried out using a restraint MD approach as used for ABCC1 transporter^55^ and initially proposed for P-gp by Wen and colleagues^63^ to ensure the different induced fit for ATP-Mg^2+^-NBD interactions. Briefly, for equilibration steps, distance between each ATP purine moiety and NBD1&2 A-loop tyrosines (namely Tyr 403 and Tyr 1043) were restrained as well as H-bonds between phosphate moieties, Mg^2+^ atoms with surrounding Walker A serine residues (Ser436 and Ser1076) and Q-loop glutamine residues (Gln477 and Gln1206) to maintain proper ATP docking in NBS. For each restraint, a harmonic potential was applied to restrain distances using ATP binding modes of close-cleft (cc) ^cc^ABCB4-(ATP)_2_ as reference. The distance restraints were applied during the thermalisation, equilibration, but also the first 10 ns of the production. They were then smoothly removed over 10 ns. The remaining part of the production run was performed without restraints. Analyses were conducted with the CPPTRAJ^64^ and plots were created using the matplotlib v3.1.1 python package. Free energy surfaces were estimated using the InfleCS method^65^. Figure rendering were carried out using the Visual Molecular Dynamics Software (v1.9.4-alpha)^66^.

### Molecular docking calculations

Direct interactions of roscovitine analogues with ABCB4 (WT and variants) were investigated using a two-step molecular docking protocol. First, sixteen snapshots from MD simulations of both WT and variant models were extracted from alchemical transformation to perform brute-force molecular dockings. Given the lack of prior knowledge regarding the interaction mechanisms of roscovitine analogues, the entire protein surface was considered as a potential binding site. The whole ABCB4 structure was divided into up to 292 search volumes, each measuring 30 × 30 × 30 Å^3^ and spaced at 10 Å intervals along each Cartesian axis. The nine selected roscovitine analogues were subjected to blind brute-force molecular dockings. For each analogue and each search volume, twenty docking replicas were systematically performed, resulting in over 50 million molecular poses. A random selection of 20% of these poses was clustered using the *cpptraj* software^64^, with k-means clustering based on the centre-of-mass coordinates of the roscovitine analogues. This process yielded ten clusters accounting for 99% of the docking poses. To incorporate transporter dynamics, each cluster identified by InfleCS from µs-scale MD simulations was considered as a target for further docking. Representative structures from these clusters were used in subsequent docking calculations. Cluster centroids were used to define the centres of new volume search spaces, and side chains of aromatic and non-polar residues within these volumes were treated as flexible. For each roscovitine analogue and volume search space, twenty additional docking replicas were conducted. Molecular docking was carried out using GPU-accelerated version, Vina-GPU^67–69^ (version 2.1), with an affinity cut-off of -4 kcal.mol^-1^ and a parallelisation scale of 8,000.

### Statistical analyses

Graphics and statistical analyses (one-way ANOVA tests) were performed using Prism version 9.00 (GraphPad software). Data are expressed as means ± standard deviation (SD). A *P* value < 0.05 was considered significant with *P < 0.05; **P < 0.01; ***P < 0.001. Symbols indicate the comparison between the control (WT or vehicle-treated) and the other tested conditions.

## Supplementary Information

Below is the link to the electronic supplementary material.

Supplementary Material 1

## Data Availability

The datasets generated and analysed during the current study are available from the corresponding author on reasonable request.
