# Supplementary material for: Molecular characterisation of the trafficking rescue of defective ABCB4 variants by roscovitine analogues
Source: Sci Rep. 2026 Feb 25;16:11031. doi: 10.1038/s41598-026-39840-6 (PMC13043924; doi:10.1038/s41598-026-39840-6)
Supplement: Supplementary file 1 — Supplementary Material 1 [file 41598_2026_39840_MOESM1_ESM.pdf]

SUPPLEMENTARY INFORMATION

--

Molecular characterisation of the trafficking rescue of defective ABCB4 variants by roscovitine analogues

Manon Banet[1,*], Veronica Crespi[2,*], Jonathan Elie[3], Yosra Riahi[1], Mounia Lakli[1], Elodie Mareux[1], Emmanuel Gonzales[1,4], Emmanuel Jacquemin[1,4], Laurent Meijer[3], Martine Lapalus[1], Florent Di Meo[2,5,§], Thomas Falguières[1,§,†]

[1] Université Paris-Saclay, Inserm, Physiopathogénèse et traitement des maladies du foie, UMR_S 1193, FHU Hepatinov, F-91400 Orsay, France.

[2] Inserm, Université de Limoges, Pharmacology & Transplantation, UMR 1248, Centre de Biologie et Recherche en Santé, F-87000 Limoges, France.

[3] Perha Pharmaceuticals, Hôtel de recherche, Presqu'île de Perharidy, F-29680 Roscoff, France.

[4] Assistance Publique - Hôpitaux de Paris, Paediatric Hepatology & Paediatric Liver Transplant Department, Reference Center for Rare Paediatric Liver Diseases, FILFOIE, ERN RARE LIVER, Faculté de Médecine Paris-Saclay, CHU Bicêtre, F-94270 Le Kremlin-Bicêtre, France.

[5] Inserm US042, CNRS UAR2015, Université de Limoges, CHU Limoges, BISCEm, F-87000 Limoges, France

* These authors contributed equally.

[§] These authors jointly supervised this work.

[†] **Corresponding author:** Thomas Falguières, PhD – UMR_S 1193 Inserm / Université Paris-Saclay – Bâtiment Henri Moissan – 17, avenue des Sciences – 91400 Orsay, France. e-mail: thomas.falguieres@inserm.fr

SUPPLEMENTARY METHODS

Cytotoxicity assays

Cytotoxicity of drug candidates was assessed by the conversion of 3-[4,5-dimethylthiazol-2-yl]- 2,5 diphenyl tetrazolium bromide (MTT; Merck/Sigma-Aldrich, Saint-Quentin-Fallavier, France) into formazan crystals by living cells, as described[1-3]. In brief, six hours after seeding into 96-well plates, HEK cells were treated with 1 to 100 µM of the selected molecules in triplicate. After 16 h of drug treatment, 0.125 mg/mL MTT (final concentration) was added in each well, and cells were re-incubated at 37°C for 2 h. Cells were then lysed in pure DMSO, and absorbance at 550 nm was measured using a Wallac Victor[3] multilabel plate reader (PerkinElmer, Massy, France). Cytotoxicity was calculated for each well and after background subtraction, means were expressed as percentages of the mean for cells treated with vehicle only.

SUPPLEMENTARY TABLE

Supplementary Table 1. Calculated free energy differences from DDMut webserver*(1)*.

Conformation	#1	#2	#3	#4	#5	#6	#7	Average
**I490T**	-2.52	-2.68	-2.49	-2.78	-2.53	-2.41	-2.80	-2.6 ± 0.2
**I541T**	-2.85	-2.65	-3.16	-2.95	-2.89	-2.85	-3.00	-2.9 ± 0.2
**L556R**	-2.50	-1.79	-2.76	-2.25	-1.48	-1.52	-2.76	-2.2 ± 0.6

Single mutation free energy differences ($\Delta\Delta G^{WT\rightarrow Mut}$) were calculated using 7 snapshots obtained from InfleCS clustering from MD simulations conducted on the ABCB4-WT MD simulations, considering both apo and ATP-bound states. Free energy differences were calculated using the protein alone.

[(1)] https://biosig.lab.uq.edu.au/ddmut/

SUPPLEMENTARY FIGURES

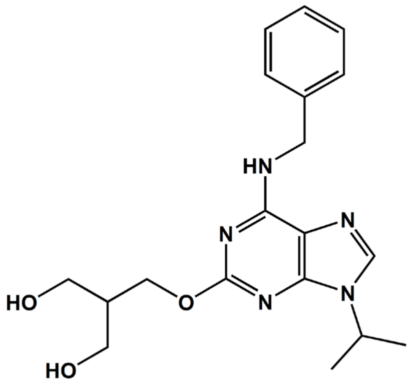

MRT2-130

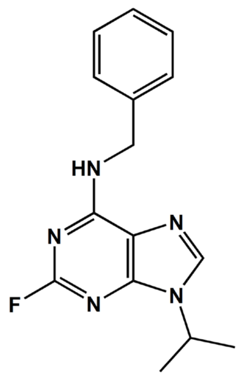

MRT2-174

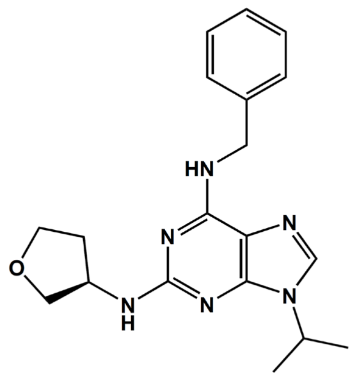

MRT2-467

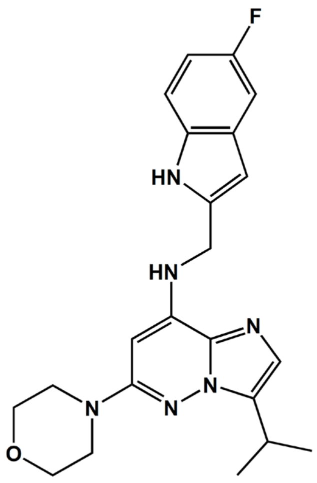

MRT8-202

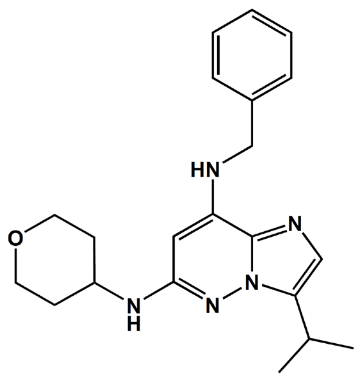

MRT8-526

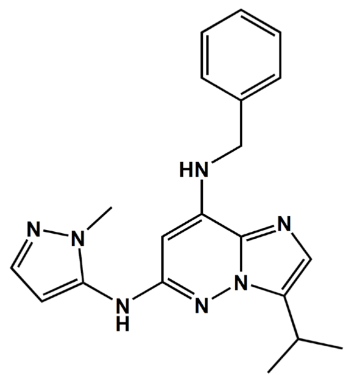

MRT8-836

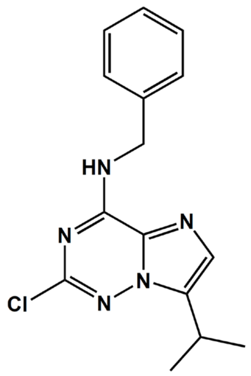

MRT13-170

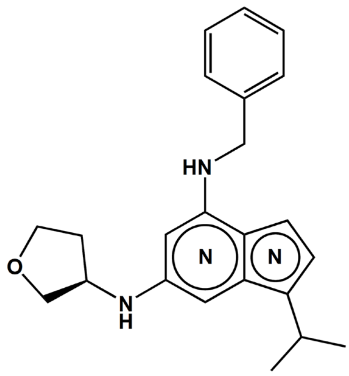

MRT14-467

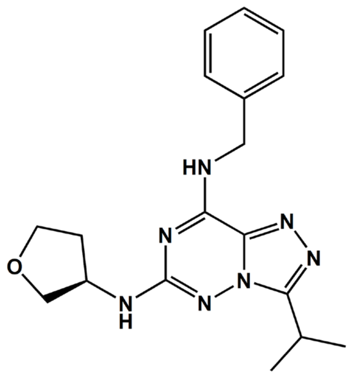

MRT16-467

Supplementary Fig. 1. Structure of the nine selected roscovitine analogues. Note that for intellectual property reasons (patent pending), the MRT14-467 core has been replaced by a generic structure.

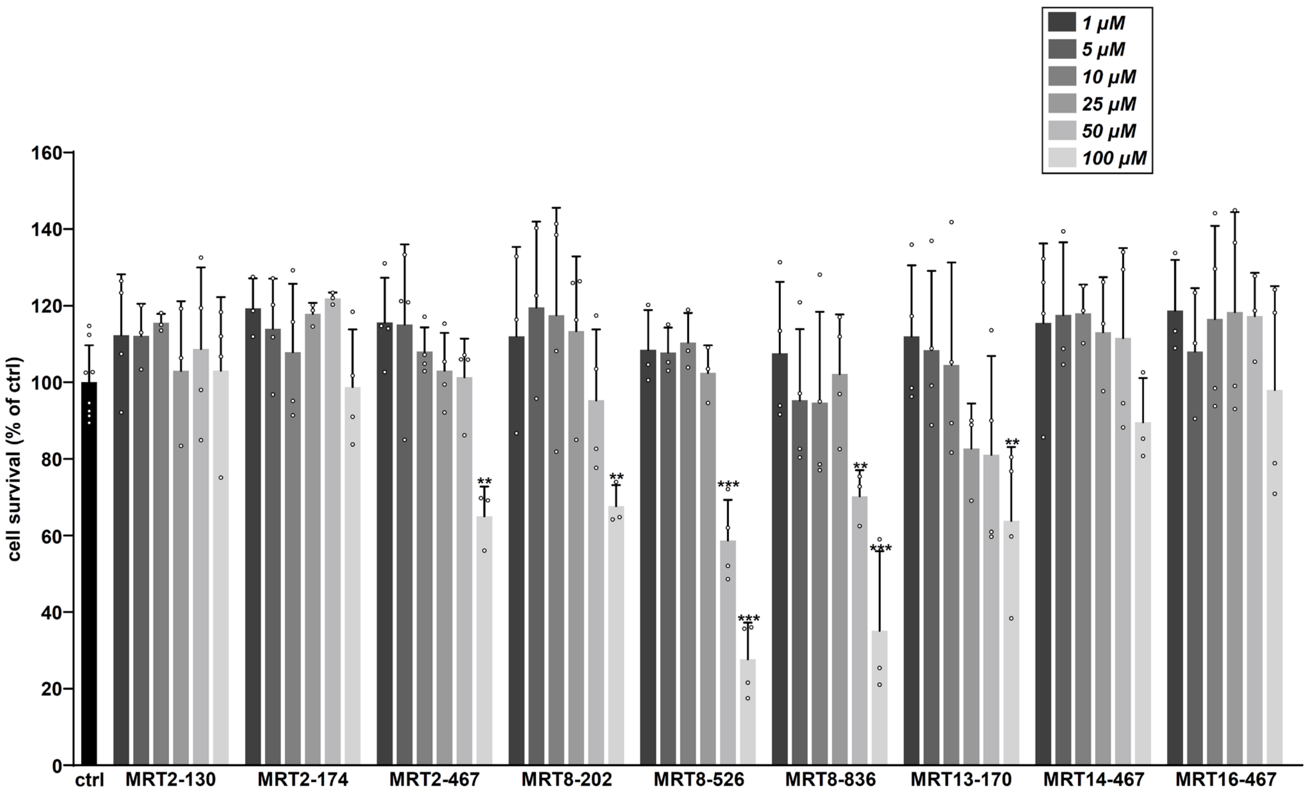

Supplementary Fig. 2. Analysis of the cytotoxicity of selected roscovitine analogues. HEK cells were treated with the indicated concentrations of selected roscovitine analogues or with the vehicle (ctrl, DMSO) for 16 hours. The cell viability was then assessed (MTT release assay) and expressed as the percentage of the mean for vehicle-treated cells. Means (± SD) of at least three independent experiments per condition are shown.

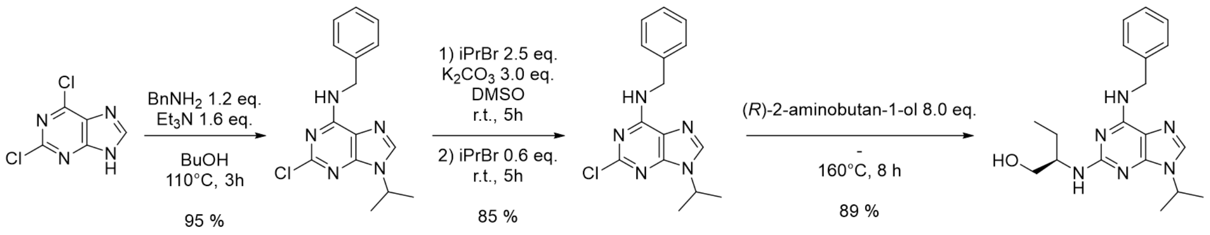

Supplementary Fig. 3. Roscovitine synthesis pathway.

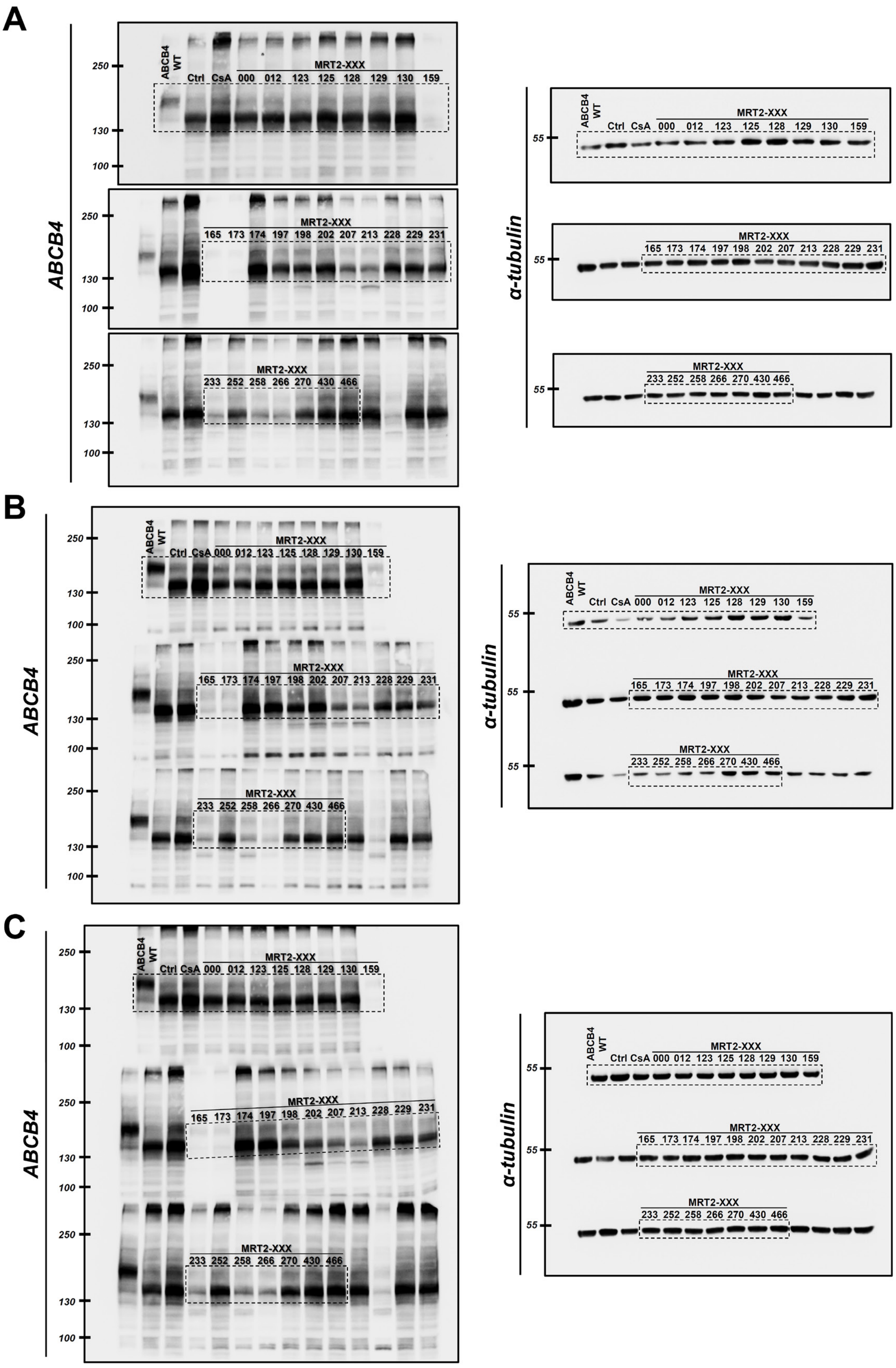

Supplementary Fig. 4. Full immunoblots related to main Fig. 2, for Fig. 2A (A), Fig. 2C (B), Fig. 2E (C). Results shown in main figures are delineated by dotted rectangles. MW (in kDa) are indicated.

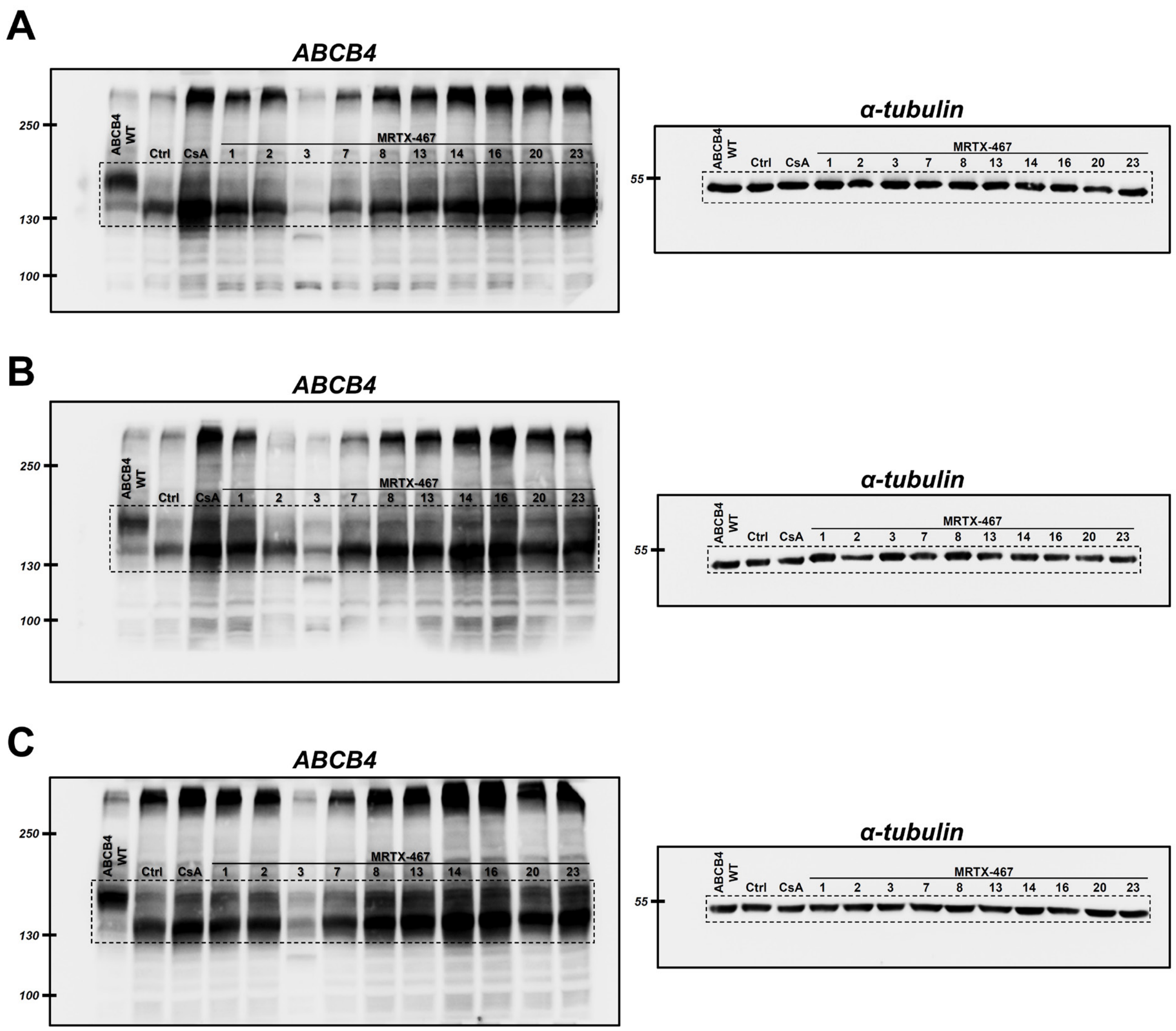

Supplementary Fig. 5. Full immunoblots related to main Fig. 3, for Fig. 3A (A), Fig. 3C (B), Fig. 3E (C). Results shown in main figures are delineated by dotted rectangles. MW (in kDa) are indicated.

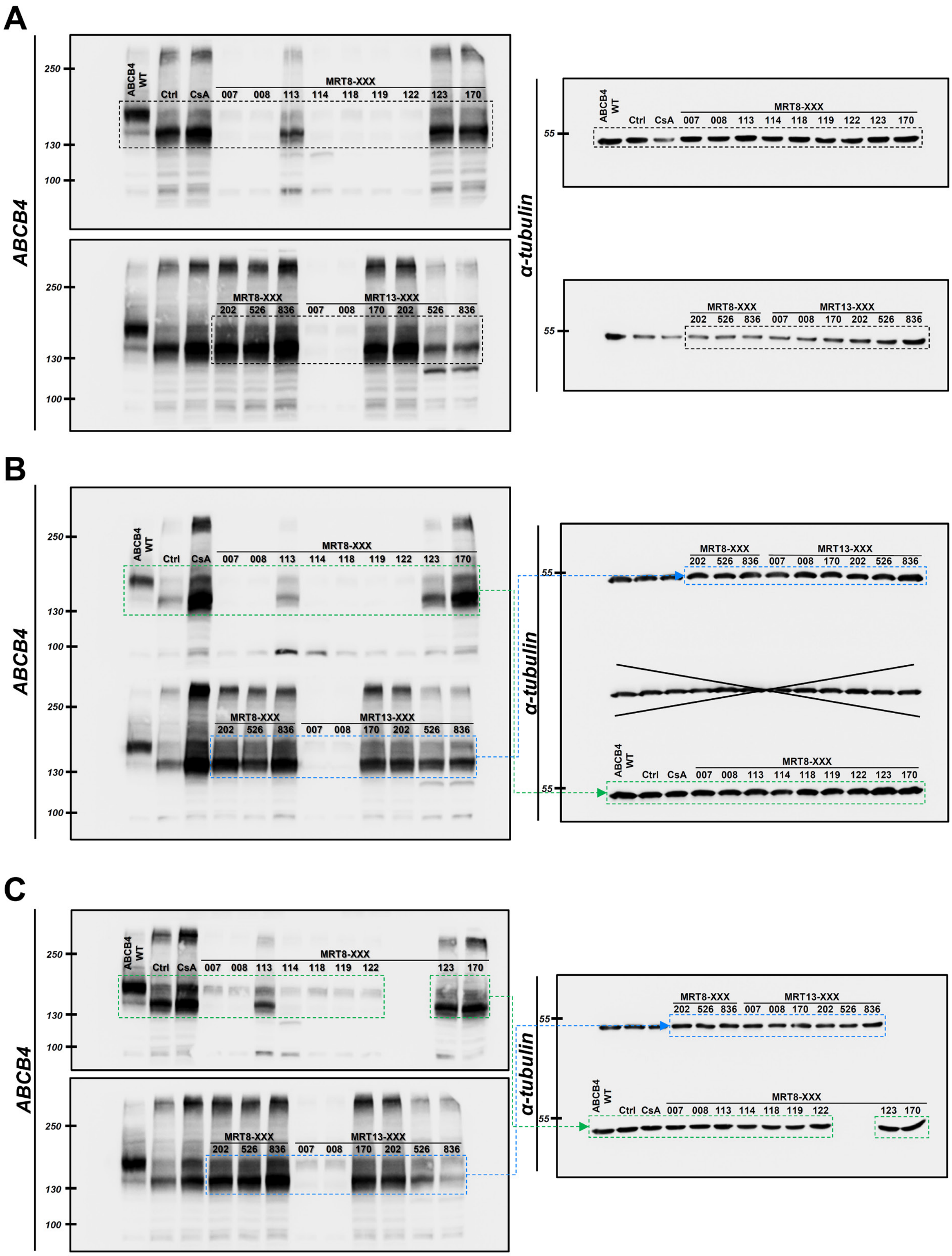

Supplementary Fig. 6. Full immunoblots related to main Fig. 4, for Fig. 4A (A), Fig. 4C (B), Fig. 4E (C). Results shown in main figures are delineated by dotted rectangles. MW (in kDa) are indicated.

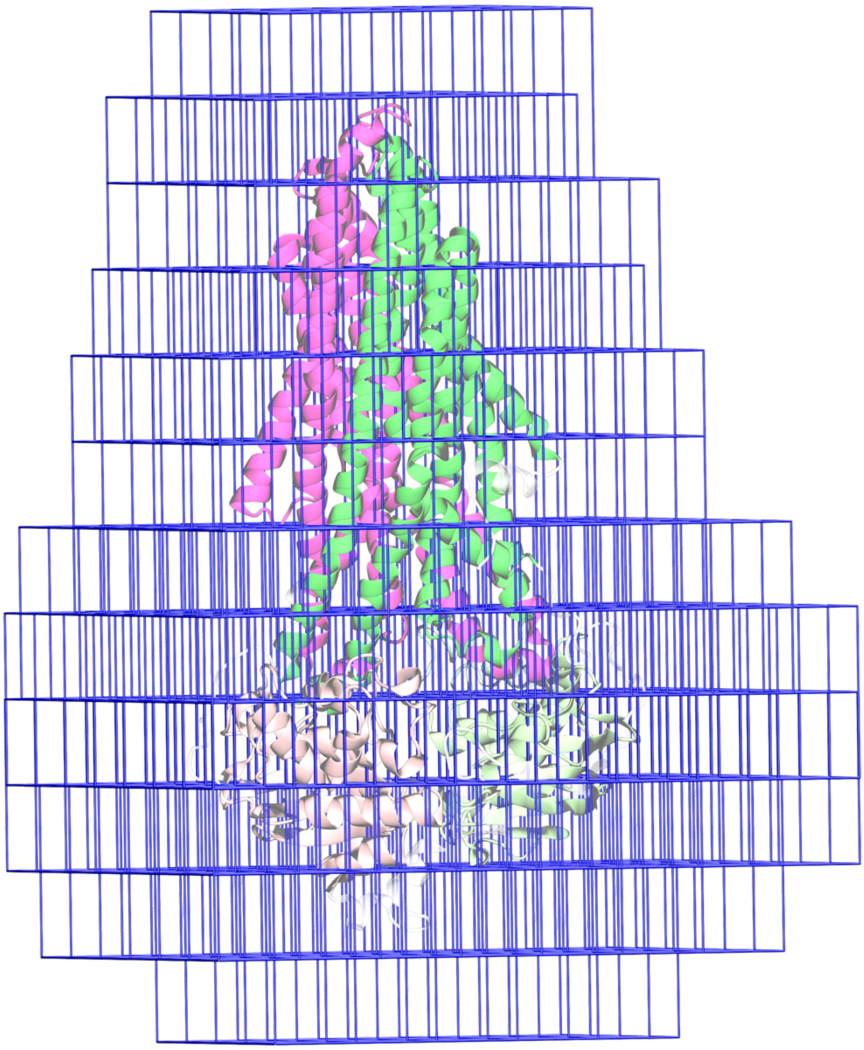

Supplementary Fig. 7. Brute-force blind molecular docking. Example of volume search spaces for apo-^{IF}ABCB4-WT models. Each volume space was set at 30x30x30Å with an overlap of 10 Å between each volume.

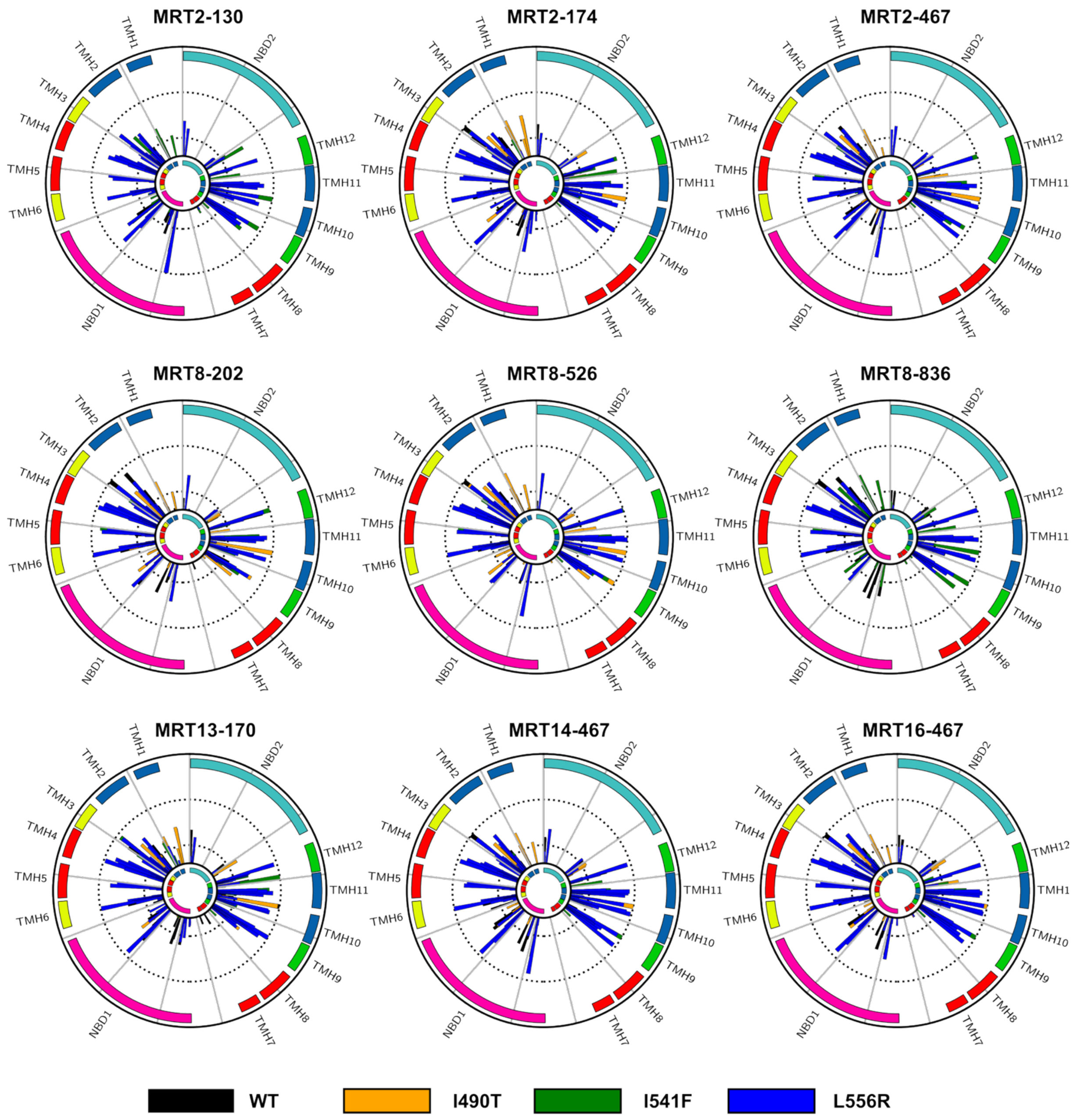

Supplementary Fig. 8. Residue-roscovitine analogue contact analyses. The most frequent contact residues obtained by considering molecular poses from refined flexible ensemble docking. Given the large variability between the number of poses, contact fractions were normalised as 1.0 for the most frequently observed residues per molecule and ABCB4 variant.

SUPPLEMENTARY REFERENCES

1 van Meerloo, J., Kaspers, G. J. & Cloos, J. Cell sensitivity assays: the MTT assay. *Methods Mol Biol* **731**, 237-245 (2011).

2 Ben Saad, A. et al. Effect of CFTR correctors on the traffic and the function of intracellularly retained ABCB4 variants. *Liver Int* **41**, 1344-1357 (2021).

3 Lakli, M. et al. Identification of new correctors for traffic-defective ABCB4 variants by a high-content screening approach. *Commun Biol* **7**, 898 (2024).
